# Simplified Procedure for General Synthesis of Monosubstituted Piperazines—From a Batch Reaction Vessel to a Flow (Microwave) Reactor

**DOI:** 10.3390/molecules25092168

**Published:** 2020-05-06

**Authors:** Dana Němečková, Eva Havránková, Jan Šimbera, Richard Ševčík, Pavel Pazdera

**Affiliations:** Department of Chemistry, Masaryk university, Kotlářská 2, Brno 611 37, Czech Republic; 80084@mail.muni.cz (D.N.); havrankova.e@mail.muni.cz (E.H.); 108324@mail.muni.cz (J.Š.); pazdera@chemi.muni.cz (P.P.)

**Keywords:** piperazine, monosubstituted, derivative, heterocyclic, catalysis, heterogeneous, microwave, synthesis, supported catalyst, reactor

## Abstract

We reported a novel simplified synthetic procedure for the preparation of monosubstituted piperazine derivatives which can now be easily prepared in a one-pot-one-step way from a protonated piperazine with no need of introduction of a protecting group. Reactions, proceeding either at room or higher temperatures in common solvents, involve heterogeneous catalysis by metal ions supported on commercial polymeric resins. A general synthetic scheme was successfully applied to afford a wide range of monosubstituted piperazines. Furthermore, we picked up a set of piperazine derivatives and studied the possibilities of microwave acceleration of given synthetic reactions to make them even more efficient. Our research proceeded from a simple batch technique to the construction of a flow microwave reactor prototype and resulted in promising findings which are summarized and discussed in the article.

## 1. Introduction

Generally, piperazine derivatives are very desirable building blocks for the production of many pharmaceuticals and they have great potential in the field of pharmaceutical research and development [1,2,3,4]. Although piperazine itself shows some significant medicinal features such as anthelmintic activity [5], piperazine or substituted piperazine moieties appear in more complex structures of both commercially available (anti-vertigo agents Cyclizine and Meclizine or drugs to treat erectile dysfunction and pulmonary arterial hypertension (PAH) such as Sildenafil or Vardenafil) and potential pharmaceuticals. The piperazine structure is present in (potential) antimicrobial agents [6], antipsychotics or antidepressants [7,8,9], pain moderators/suppressors [10] or drugs for treatment of HIV [11,12] or neurodegenerative diseases such as Parkinson’s or Alzheimer’s disease [13], and many anti-cancer agents contain substituted piperazine moiety as well [3,14,15]. 

An introduction of a piperazine structural motive into the drug molecule is usually realized via 1-monosubstituted piperazine and, thus, there is still an unrelenting demand for these kinds of piperazine derivatives in the field of pharmaceutical research and development. However, their broader use in the pharmaceutical industry is still limited by their high price, which is a consequence of their problematic preparation. In general, known methods mostly suffer from low yields because of multi-step synthesis, competitive reactions or complicated isolation and purification, and often environmentally inappropriate or expensive agents and solvents are employed. When the direct monosubstitution of a free piperazine is involved, the yields of monosubstituted piperazines are significantly decreased by the occurrence of competitive reactions leading to disubstituted derivatives [16,17]. Nonetheless, over the last few years, some improved procedures based on a simple reaction of a free piperazine with halogenated reagents were published. Although yields were somehow improved, syntheses still feature some particular issues such as the usage of chlorinated solvents [18,19,20], multistep isolation/purification process often including column chromatography [18,19,21,22,23,24,25] or disadvantageous atom economy when the usual excess of piperazine must be used [3,19,23,24,25]. 

In case of some benzyl- and substituted benzyl derivatives, especially fluorinated, above-mentioned issues were suppressed and monosubstituted products were obtained in very high yields [20,26,27,28,29,30,31,32]. However, the same reaction scheme does not usually work sufficiently well in the case of other substituted benzyl derivatives (yields are usually significantly decreased). It is important to add that the described reaction procedure has seemingly not been successfully applied to the synthesis of other monosubstituted piperazines apart from those benzyl derivatives, because only a few mentions were found in literature. However, preparations are, again, encumbered by usual issues such as low yields and the abundant formation of competitive disubstituted derivatives [33], and in those cases, alternative ways of synthesis are preferred.

The generally acceptable and widely used method to prepare monosubstituted piperazines is based on the employment of protecting groups such as tert-butyloxycarbonyl (Boc) or similar alkoxycarbonyl, acyl or benzyl groups [34,35,36,37]. The main disadvantage of such approach is the multi-step synthetic procedure. First, a protecting group must be bound to one of the piperazine nitrogen atoms. The resulting intermediate subsequently reacts through the second nitrogen atom with the appropriate reagent, and finally a protecting group is removed to yield monosubstituted piperazine derivative. However, the multi-step synthesis is reflected in decreased overall yields and the high price of resulting products, which is the reason why this way is avoided in larger scale production. 

Another approach to obtain monosubstituted piperazines is based on Michael addition. Secondary amines are often used, due to their higher nucleophilicity in aza-Michael additions with a variety of unsaturated compounds, and therefore some examples involving the addition of a free piperazine onto unsaturated compounds (acrylonitrile, methyl acrylate etc.) can be found in literature [38,39,40]. Reactions usually proceed with decent yields, and monosubstituted—as well as symmetrically disubstituted—piperazine derivatives can be prepared intentionally [41]. On the other hand, reactions require the presence of a catalytic system, mostly based on copper or cerium, and some additional activation of an unsaturated reagent is preferred to obtain higher yields. Moreover, a competitive reaction leading to a disubstituted product can be an issue when monosubstituted piperazine is intended to be prepared using aza-Michael addition [38,42].

Some alternative synthetic methods can be found in literature [43,44,45].

As most of the above-mentioned articles deal with the synthesis of potential novel drugs incorporating piperazine moiety, it is obvious that there is still a high demand for monosubstituted piperazine derivatives and a challenge for the development of new, simple and inexpensive methods of their synthesis and manufacturing. 

In recent years we have published a number of reports and patents on a simplified one-step synthesis of a variety of monosubstituted piperazine derivatives [46,47,48,49,50] which are based either on the aza-Michael addition of unsaturated compounds onto piperazine or on the electrophilic substitution of piperazine with a suitable chloroderivative.

Although a simplified synthetic methodology for piperazine derivatives was published, we still intensively studied other ways to accelerate and further simplify the described procedures, especially regarding potential commercial manufacturing on a larger scale. We focused on the utilization of microwave (MW) radiation, as microwave processes are known to be environmentally friendly, enabling energy saving. Other benefits obtained in chemistry consist of increased reaction rates, the reduction of side-products, and the improvement of the product purity compared to conventional heating [51]. Preliminary experiments involving a batch MW assisted technique showed promising results, but we continued further, and particularly for larger-scale experiments–we proposed and verified an idea of a flow reactor equipped with a small MW unit to replace conventional heating. The aim of the research and development was to introduce a flow MW reactor prototype that can be employed for the preparation of monosubstituted piperazines with the prospect for its utilization for a broader scale of reactions. From the very beginning, it was assumed to be operated at conditions close to standard ones, especially to avoid operating at high pressures, because the construction of pressurized reactors necessitates the use of expensive advanced materials (stainless steel, PTFE, coated materials, etc.). Moreover, a higher pressure requires more emphasis on safety measures. Additional demands on a prototype consisted of the use of a heterogeneous catalyst in the flow system. To summarize all the requirements, we aimed to propose a cheap, simple and robust MW flow reactor prototype for general organic synthesis, capable of larger volume processing. The resulted proposal should contribute to the actual state of art in the field of MW flow reactors with a different approach. Nowadays, various batch MW reactors are available for organic synthesis [51,52], ranging from a simple MW oven-based laboratory system to advanced MW batch reactors offered by Biotage [53], Anton Paar [54] or Milestone [55], however, they are limited by a volume that can be processed in a single batch. Flow MW reactors are usually employed only in laboratory practice for the preparation of small quantities, and they are usually realized as a small diameter tube or multiple capillary reactors which are exposed to microwaves in a single- or multimode MW cavity [56,57]. Reactions are usually performed at high pressures and temperatures. The expansion of MW flow reactors into large scale processing or commercial use is still limited [52], probably due to their complicated arrangement and high price.

## 2. Results and Discussion

### 2.1. One-step Synthesis of Monosubstituted Piperazine Derivatives

**Scheme 1:**
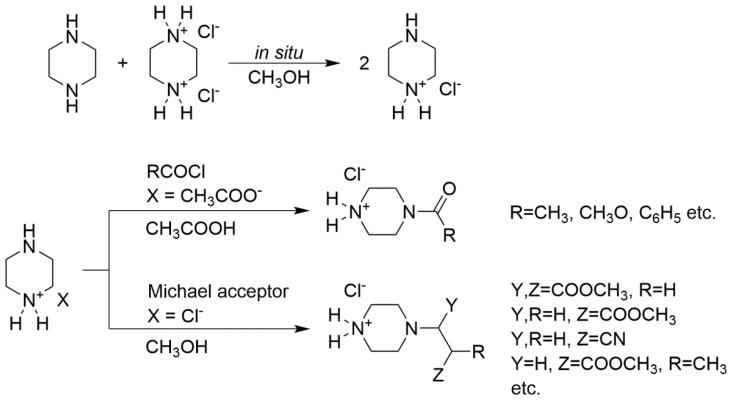
A general reaction scheme of synthesis of monosubstituted piperazine derivatives.

As it was described in patented procedures [46,47,48] and literature [49,50], we discovered and verified a new and simple one-pot-one-step synthetic procedure for the preparation of a large variety of monosubstituted piperazine derivatives which can now be obtained in high yields and purity while keeping costs low. The principle of the synthetic procedure (Scheme 1) is based on a reaction of in-situ formed piperazine-1-ium cation (in a form of monohydrochloride or monoacetate) with an appropriate reagent, such as acyl reagents (chlorides, anhydrides, etc.) or Michael acceptors (activated alkenes, alkynes) or others. The protonation of piperazine is employed as a simple way of protection of the secondary nitrogen atom resulting in the suppression of a competitive reaction leading to disubstituted derivatives. Although the principle of a protonated piperazine was rarely mentioned in literature [26,27,28,32], it was not applied in a broader scale for the synthesis of other monosubstituted piperazines. Nonetheless, our long-time research has shown that the principle is applicable to the synthesis of a wide range of monosubstituted piperazines. Acid/base equilibria of piperazine and some of its derivatives were studied, discussed, and published formerly [49,50] and they are considered to be an essential aspect influencing the course of the reactions. It was also discovered [46,47,48,49,50] that reactions of a protonated piperazine with different reagents are strongly influenced by metal ions which significantly accelerate the course of reactions. In particular, Cu(II) and Ce(III) ions proved to be effective catalysts for the reactions of a piperazine with electrophilic and Michael reagents because they activate reactants with lower reactivity. Disadvantages of a homogeneous catalysis were then surpassed by supporting metal ions onto polymeric resins [49,58]. As a support for metal catalysts, we used cheap, commercially available ion-exchanger polymeric resins, especially resins of a weakly acidic macroporous polyacrylate type. The supporting of metal onto polymeric resin is simply performed by treatment of a polymeric resin with a solution of a relevant metal containing compound. Supported metal ions evidence the same catalytic activity but heterogeneous catalysts can be easily separated from a reaction mixture by filtration, and when washed thoroughly, they can be reused several times without a loss of activity.

Reactions proceed easily in usual solvents, such as methanol or acetic acid, at room temperature or under reflux. The starting piperazine hydrochloride/acetate can be simply prepared on the dependence of a solvent used. When acetic acid is involved as a solvent, then piperazine monoacetate is formed when a free piperazine is dissolved. When using methanol, the reaction of a free piperazine with piperazine dihydrochloride yielding piperazine monohydrochloride is carried out as the first step. In fact, piperazine dihydrochloride primarily acts as an auxiliary substance, and when the reaction is completed, it is recovered from the reaction mixture and it can be reused when washed properly. Reactions proceeding under the above-mentioned conditions provide desired monosubstituted piperazine derivatives in high yields and purity. The formation of corresponding symmetrically disubstituted by-products is suppressed by the set conditions (especially by the molar ratio of a free piperazine and piperazine dihydrochloride) and by the nature of the second reactant which influences besides other things the basicity/nucleophilicity of the second nitrogen atom of piperazine in a formed monosubstituted piperazine derivatives [49,50]. Despite the above-mentioned facts, disubstituted derivatives are formed in variable amounts during the reactions, but they are easily separated when the main products are isolated and their traces are then removed during recrystallization. More details about preparations and the overall background of the procedure can be found in patents and literature [46,47,48,49,50]. 

The described synthetic arrangement, based on a reaction of piperazine-1-ium cation, provides a simple, easy, and cheap route to monosubstituted piperazine derivatives while respecting the principles of green and sustainable chemistry. Reactions are catalyzed by metal ions supported on a commercial polymeric resin to shorten reaction times. The heterogeneous catalyst is easily separated from the reaction mixture and can be further reused. A general synthetic procedure was successfully applied to afford a variety of monosubstituted piperazine derivatives which are easily isolated in high yields (see Table 1, procedure **A**) and purity. These aspects, together with low costs, may be considered to be crucial for their greater utilization in pharmaceutical research and development. Some of the compounds are now commercially available for research and development via the websites www.entwickchemicals.com and www.fichema.cz. 

### 2.2. Microwave Assisted Synthesis of Monosubstituted Piperazine Derivatives

Since most of the reactions proceed under reflux for several hours, we focused on the utilization of microwave irradiation to replace conventional heating. First of all, we decided to set up simple batch experiments using a slightly modified commercial microwave oven. To enable the usage of a water condenser or input/output hosepipe, a small hole (reinforced by a steel sheet grounded to chassis) was drilled through the upper wall of the oven. A series of reactions was then selected for the comparison of different synthetic procedures: the classic flask procedure (denoted as **A**), microwave assisted batch process (**B**) and a process employing a flow microwave reactor prototype (**C**). 

In the case of setup **B**, a reaction flask, containing a reaction mixture together with a supported catalyst, was placed into the oven and a glass adaptor, passing through the hole in the upper wall of the oven, was attached. Subsequently, a water condenser was mounted on the top of a glass adaptor to be out of the reach of microwaves. 

**Scheme 2:**
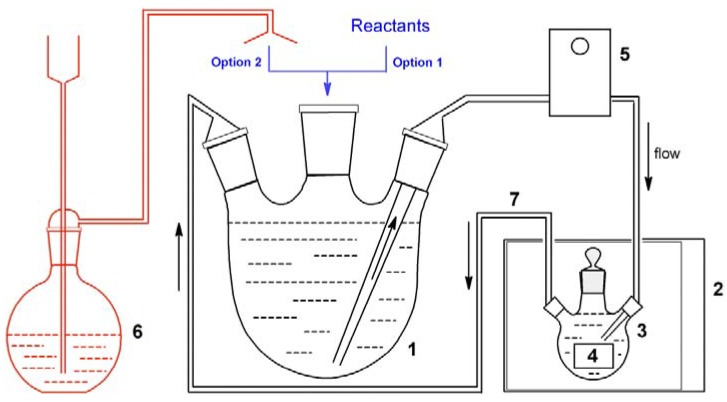
Scheme of a functional prototype of a flow microwave reactor used for the procedures **C**. (1) reservoir flask, (2) commercially available microwave oven, (3) reaction flask, (4) catalytic bed, (5) pump, (6) safety flask, (7) silicone tubing.

The first set-up of a microwave flow reactor prototype (procedure **C**) is presented in Scheme 2. When employing a flow reactor, a solution containing piperazine-1-ium cation is first poured into the reservoir flask (1) through the third neck (Option 1). A supported metal catalyst is loaded into a small three-neck reaction flask (3) placed in the MW oven (2). A catalyst is with advantage closed in a catalytic bed (4), not to be carried away from the reaction flask by a circulating mixture. A catalytic bed was realized simply by putting beads of a supported catalyst into a closed porous pouch made of polypropylene. At the advanced level, a catalytic bed can be realized as a casing made of highly porous sintered glass material. An input and output hosepipe, connecting the reaction flask with other parts, is passed through the hole in the upper wall of the oven. A silicone tubing (7) was used to connect all the parts into a closed circuit. After this, the pump of a membrane or peristaltic type (5) is switched on and the entire flow circuit (drawn in black color on Scheme 2) is filled with a solution containing piperazine-1-ium cation. Subsequently, the second starting compound is added into the circulating solution using the third neck of a reservoir flask (Option 1). A portion-wise addition of a compound is recommended to avoid a possible turbulent reaction which may occur when starting compounds are mixed together. A reservoir flask is then attached (Option 2) to a safety auxiliary system (drawn in red color on Scheme 2) including safety flask (6), balancing the pressure fluctuation in a flow circuit. After this, the MW oven is to be switched on to initiate the reaction. When using a MW oven, the MW power was always decreased and applied using a pulse mode to keep a reaction mixture in a reaction flask only slightly boiling. The MW irradiation was finished when the conversion of a reaction was full or highest, as it was monitored by thin layer chromatography (TLC). However, the flow reactor can be operated in a simple flow mode when MW heating is not required, as it was in the case of the reaction of methyl chloroformate. The reaction proceeds at room temperature when the reaction solution flows through the reaction flask equipped with a catalytic bed (MW heating was switched off during the course of the reaction). Similarly, only microwave heating without a catalytic bed can be used in the case of uncatalyzed reactions. 

### 2.3. Comparison of Synthetic Techniques

A series of synthetic reactions leading to monosubstituted piperazine derivatives was selected to compare classic synthetic techniques with microwave assisted syntheses. All the reactions follow Scheme 1 and, except the reaction of piperazine with methyl chloroformate, they proceed in methanol under reflux. Many experiments, when varying molar ratios, supported catalysts or other variables were carried out and the best results of the experiments employing different synthetic techniques are summarized in Table 1.

Products obtained by MW assisted techniques (**B**-**C**) were routinely identified using ^1^H and ^13^C-NMR, melting point determination and FT-IR measurements and data were compared with those belonging to products obtained by classic flask method (**A**) (Supplementary Materials). A comparison showed that the same monosubstituted piperazine derivatives were prepared regardless of the involved synthetic procedure (**A**-**C**). Moreover, products were obtained in the same purity as well, i.e., exceeding 98.0% (based on ^1^H NMR measurements), when recrystallized according to described procedures (average yields given in Table 1 are related to recrystallized products). When in doubt about the purity of the products, LC-MS experiments were performed to confirm purity determination by ^1^H-NMR. The obtained results usually showed a pure product (purity ~ 100%) or only traces of impurities not exceeding 2% (usually around 1%) were observed. Although impurities are usually easily removed during isolation of the main product and subsequent recrystallization (especially piperazine dihydrochloride or unreacted piperazine), some traces may remain. These are comprised mostly of symmetrically disubstituted by-products which may be formed concurrently in the dependence of a reagent used, but when keeping molar ratios shown in Table 1, their formation is significantly decreased. 

The obtained results further confirmed our expectation regarding the effect of MW irradiation on the course of reactions. It can be seen that procedures proceeding under MW irradiation (**B**-**C**) show the same results concerning yields and purity, but reactions have the advantage of finishing in considerably shortened times, mostly several times shorter. Particularly in the case of reactions where all of three synthetic techniques were studied, it is obvious that reaction time is significantly decreased when proceeding from a classic flask method (**A**) through a batch process (**B**) to a flow microwave reactor processing (**C**). The reason can be found in an active part of the flow reactor—A small reaction flask in the MW oven. A reaction flask (volume of 100–150 mL) always contains only a fraction of a reaction solution, while the majority of a volume is always located in the reservoir flask (volume of 1–2 L). Thus, only a small volume of a reaction mixture is treated with MW radiation, and simultaneously, it is affected by a hyper-molar amount of a supported catalyst loaded in a catalytic bed. Under these conditions, reactions can proceed very quickly. The amount of a catalyst can be thus considered independent on the total volume of a reaction mixture. Additionally, a small volume of a solution in the reaction flask then requires only a low microwave power, i.e., a commercially available microwave oven (or a MW unit with a similar power) instead of a huge and expensive device for large volume processing. A reservoir flask can, in fact, be of any volume and any volume of a solution (10–12 times larger than volume of a reaction flask in a current set-up) can be processed using the proposed principle of the flow reactor. Of course, some proportion between volumes of reservoir and reaction flask should be maintained not to extend reaction times unduly. Employing the set-up described in Scheme 2, we successfully processed reaction mixture volumes yielding between 15–55 g of crude products (particular experiments were repeated several times). 

Nonetheless, problems may occur when some intermediate or by-product starts to crystalize from a solution during reaction because it can be deposited somewhere in the circuit or it can cause a pump malfunction. Optionally, a solid particle filter should be placed into the system to prevent leakage of solids from a reaction flask or, on the contrary, an exchangeable filter can be used to separate solid (by-)products from a solution. Of course, a problem may remain when a large quantity of a solid intermediate/by-product is formed. The flow system may then collapse, despite the filter employed as solid mass forming a stopper to stop the running flow. The employment of a flow reactor is not recommended for this type of reaction. On the other hand, a small quantity of a solid in a flowing mixture is not considered an issue, as it occurred during some of the experiments.

### 2.4. A Flow Reactor with a Microwave Unit and/or Catalytic Bed

**Figure 1:**
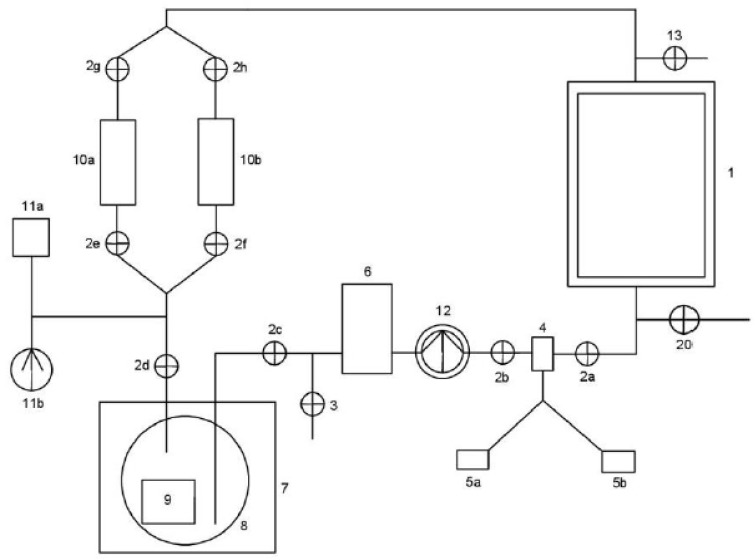
Patented principle of a flow reactor with a microwave unit and/or catalytic bed, scheme taken from [59], inner arrangement of a modular reservoir is not shown. Annotations are as follows: 1–modular reservoir, 2—valves, 3—outlet valve, 4—septum, 5a—inlet point, 5b—sampling point, 6— pump, 7—microwave unit, 8—reaction vessel, 9—catalytic bed, 10—exchangeable filters, 11a—manostat, 11b—pressure gauge, 12—flowmeter, 13—outlet/venting valve, 20—auxiliary inlet/outlet valve.

In consequence with the performed experiments, we focused on the design of a potential commercially available flow MW reactor, reflecting obtained results and fixing observed issues. We successfully introduced and patented a utility model of a flow reactor with a microwave unit and/or catalytic bed [59]. A flow reactor (Figure 1) can be operated in three working modes: (i) microwave only mode for employment of polar or polarizable reagents, (ii) microwave mode with catalytic bed when synergy of both of the processes is necessary and (iii) catalytic bed only mode, where a catalyzed reaction proceeds at room temperature and no microwave acceleration is needed. The proposed set-up is not intended to work under higher pressure and thus its construction can be realized using cheap and easily available materials (polypropylene, silicone, glass, etc.) requiring no additional coatings or finishes. A proposed design of a flow reactor is variable and can be adjusted for a variety of volumes. It is assumed to be utilized for the processing of 2–200 L of a mixture using a reaction vessel of volume of 0.2–1 L. Moreover, it can be combined with an ultrasonic flow reactor, which was presented recently and described in patent [60]. Nowadays, we are searching for a partner to finish the design and, in particular, verify the functionality and overall safety of the flow microwave reactor prototype, hopefully, to be offered for commercial use. Although the principle of a proposed flow reactor was successfully verified by a number of experiments on a smaller scale (up to ca. 55 g of a crude product), in the case of the flow MW reactor for commercial production on a larger scale, some of the features have to be re-arranged because a lot of aspects must be taken into account to ensure uniform heating, constant conditions and reproducibility [51]. Moreover, the reactor has to comply with safety requirements. Nonetheless, the main advantage of a proposed flow system—a division into a large reservoir part (always containing a majority of a processed mixture) and a small volume active part (where reaction proceeds under MW irradiation and/or catalysis)—can help to solve some expected issues (uneven distribution of microwaves in a larger space, loss of microwave power, safety, etc.) because only a low volume, low power microwave unit is necessary, even in the cases where large volumes will be processed. On the other hand, the utilization of a sophisticated (laboratory) microwave unit (with a focused microwave beam, even distribution of microwaves, etc.) can bring further benefits, such as shortened reaction times, better yields, and more comfortable handling. Of course, large-scale processing using an advanced model of the flow MW reactor intended for commercial use will certainly require extensive testing and verification.

## 3. Materials and Methods 

All reagents were purchased from commercial suppliers (Sigma-Aldrich, Acros Organics. Missouri, MS, USA) and were used without further purification. Solvents were purified according to the standard methods [61] before use, when necessary.

All the syntheses follow procedures which are described in literature [46,47,48,49,50], although the molar ratios of starting compounds had to be altered slightly in some cases because of decreased yields or due to the excessively increased occurrence of disubstituted by-products. Some additional improvements of literature methods are comprised in the below-described procedures. Molar ratios, supported metal catalysts, reaction times and yields are shown in Table 1. The supported catalyst is always recovered from a reaction mixture after completion of a reaction, washed thoroughly with methanol and dried. It can then be reused. Piperazine dihydrochloride isolated from a reaction mixture can be processed in the same way and reused as well.

*General procedure for preparation of a supported catalyst*: Supported catalysts were prepared according to patented procedures [49,58]. Purolite C 104 Plus (Purolite Worldwide, weakly acidic macroporous cation-exchanger of polyacrylate type, ionic form H^+^, total volume capacity 4.5 mmol/cm^3^, specific gravity 1.19 g/cm^3^) was suspended in water and a saturated aqueous Na_2_CO_3_ solution was added portion-wise under stirring until the pH of the solution remained at value of 12 for 10 min after the last addition. After this, the aqueous solution was decanted and resin beads were washed thoroughly by water. A water solution of a relevant metal containing compound was prepared (Ce(III): Cerium(III) nitrate hexahydrate, Cu(II): copper(II) acetate hydrate, Cu(I): copper(II) nitrate trihydrate + aqueous NH_3_), and poured onto resin beads. The resultant solution was stirred overnight. (In case of supported Cu(I) catalyst, an additional step follows: a solution was decanted and resin beads were washed properly with water; then a water solution of NH_2_OH:HCl was added to reduce Cu(II) ions to Cu(I) ions) Subsequently, the aqueous solution was decanted again and resin beads were washed 2 times by water and subsequently 2 times by methanol and finally dried in vacuum to constant weight. 

*Classic flask syntheses* (**A**): at first, a solution containing active piperazine-1-ium cation (piperazine monohydrochloride or monoacetate) was prepared in situ either from equimolar amounts of anhydrous piperazine and piperazine dihydrochloride hydrate in methanol (10–20 mL of methanol per 1 g of anhydrous piperazine, a solution can be heated to complete dissolution of solids) or by dissolving anhydrous piperazine in acetic acid (8 mL of glacial acetic acid per 1 g of anhydrous piperazine, temperature was maintained below 40 °C) in the case of a reaction of methyl chloroformate. After this, a corresponding reagent was added dropwise into a stirred solution at room temperature to avoid a possible turbulent reaction which may occur when starting compounds are mixed. Finally, the supported catalyst was added (0.1 g of a supported catalyst per 1 g of anhydrous piperazine). After this, a reaction mixture was stirred at room temperature in the case of reaction of methyl chloroformate or under reflux in other cases, until the conversion of a reaction was full or highest as it was monitored by TLC.

*Batch microwave assisted experiments* (**B**) follow classic flask procedures closely. Reaction mixtures were prepared in the very same way and a relevant supported catalyst was added in amount of 0.05: 1 mol. with respect to a corresponding reagent. Subsequently, the flask was put into the microwave oven and equipped with a glass tube adaptor and a reflux condenser. After this, MW irradiation was employed instead of regular heating (reflux). Microwave oven power was always set to a minimal energy (10% of a maximal power, i.e., 80 W) and then applied using a pulse mode (typically 3 sec. of a set power then pause for 4 sec.) to keep the reaction mixture in the flask only slightly boiling. 

*Microwave flow reactor experiments* (**C**) started in the same manner and thus at first a solution of piperazine monohydrochloride or piperazine monoacetate was prepared in the same way and poured into a reservoir flask of a flow reactor. Subsequently, a catalyst (0.5: 1 mol. with respect to a corresponding reagent) was loaded into a reaction flask placed in a MW oven (a detailed scheme is described on Scheme 2). Then pump was switched on to run the flow slowly (approx. 2–5 mL.s^−1^). A corresponding reagent was added portion wise (possibility of a turbulent reaction) into the reservoir flask to be introduced into the reaction mixture. The microwave oven power was always set to a minimal energy (10% of a maximal power, i.e., 80 W) and then applied using a pulse mode (typically 3 sec. of a set power then pause for 4 sec.) to keep the mixture in the reaction flask only slightly boiling. 

*Isolation and purification* of crude products was then performed in the same way for a given monosubstituted piperazine regardless of a used synthetic method (**A**–**C**):

*(1) Methyl piperazine-1-carboxylate hydrochloride*: white crystalline solid, m.p. = 160–161 °C; ^1^H NMR (ppm, CDCl_3_): 3.22 (4H, m, 2*CH_2pip_), 3.74 (3H, s, OCH_3_), 3.83–3.86 (4H, m, 2*CH_2pip_), 9.98 (2H, bs, NH_2_^+^); ^13^C NMR (ppm, CDCl_3_): 40.62 (2*CH_2pip_), 43.18 (2*CH_2pip_), 53.23 (OCH_3_), 155.22 (C=O); FTIR (cm^−1^): 2940, 2923, 2861, 2818, 2792, 2775, 2752, 2636, 2626, 2604 (ν, C-H), 2705, 2471 (ν, NH_2_^+^), 1695 (ν, C=O), 1150 (ν_as_, C-O-C), 1044 (ν_s_, C-O-C); LC-MS (*m*/*z*): [C_6_H_13_N_2_O_2_]^+^ = 145.0972.

The reaction mixture was cooled down to 5 °C and precipitated piperazine dihydrochloride was filtered out (together with the catalyst). The solvent was then evaporated and the product was precipitated using ethyl acetate. The crude product was then recrystallized from isopropyl alcohol with addition of a charcoal. 

*(2) Methyl 2-(piperazin-1-yl)ethanoate hydrochloride*: white crystalline solid, m.p. = 156–157 °C; ^1^H NMR (ppm, CDCl_3_): 2.92–2.95 (4H, t, 2*CH_2pip_), 3.27–3.30 (6H, m, 2*CH_2pip_ + CH_2_), 3.73 (3H, s, OCH_3_), 9.74 (2H, bs, NH_2_^+^); ^13^C NMR (ppm, CDCl_3_): 43.43 (2*CH_2pip_), 49.31 (2*CH_2pip_), 51.86 (OCH_3_), 58.42 (CH_2_), 169.98 (C=O); FTIR (cm^−1^): 2972, 2943, 2918, 2726 (ν, C-H), 2821, 2788 (ν, NH_2_^+^), 1739 (ν, C=O), 1559, 1307 (δ, CH_2_), 1466, 1389 (δ, CH_3_), 1174 (ν_as_, C-O-C), 1051 (ν_s_, C-O-C); LC-MS (*m*/*z*): [C_7_H_15_N_2_O_2_]^+^ = 159.1128. 

The reaction mixture was cooled down to 5 °C and precipitated piperazine dihydrochloride was filtered out (together with the catalyst). The solvent was then evaporated to dryness and the residue was recrystallized from isopropyl alcohol with addition of a charcoal to yield the pure product.

*(3) Methyl 3-(piperazin-1-yl)propanoate hydrochloride*: white crystalline solid, m.p. = 118–119 °C; ^1^H NMR (ppm, CDCl_3_): 2.44–2.49 (2H, t, CH_2_), 2.73–2.79 (6H, m, 2*CH_2pip_ + CH_2_), 3.18–3.22 (4H, t, 2*CH_2pip_), 3.67 (3H, s, OCH_3_), 9.52 (2H, bs, NH_2_^+^); ^13^C NMR (ppm, CDCl_3_): 32.13 (CH_2_), 43.49 (2*CH_2pip_), 49.30 (2*CH_2pip_), 51.71 (CH_2_), 53.06 (OCH_3_), 172.26 (C=O); FTIR (cm^−1^): 2977, 2961, 2934, 2906, 2772, 2712, 2683 (ν, C-H), 2815, 2792 (ν, NH_2_^+^), 1727 (ν, C=O), 1558, 1302 (δ, CH_2_), 1454, 1390 (δ, CH_3_), 1171 (ν_as_, C-O-C), 1054 (ν_s_, C-O-C); LC-MS (*m*/*z*): [C_8_H_17_N_2_O_2_]^+^ = 173.1285.

The reaction mixture was cooled down and precipitated piperazine dihydrochloride was filtered out (together with the catalyst). Filtrate was then concentrated to approx. 1/3 and placed into refrigerator overnight. A precipitated fraction of piperazine dihydrochloride was filtered out and solvent was then evaporated to dryness and the residue was recrystallized from isopropyl alcohol with addition of a charcoal. The pure product was finally washed with cold acetone and dried.

*(4) 1-(4-Methylbenzyl)piperazine hydrochloride*: white crystalline solid, m.p. = 178–179 °C; ^1^H NMR (ppm, CDCl_3_): 2.34 (3H, s, CH_3_), 2.76 (4H, m, 2*CH_2pip_), 3.22 (4H, m, 2*CH_2pip_), 3.53 (2H, s, CH_2_), 7.12–7.19 (4H, dd, 4*CH_arom_), 9.53 (2H, bs, NH_2_^+^); ^13^C NMR (ppm, CDCl_3_): 21.06 (CH_3_), 43.58 (2*CH_2pip_), 49.39 (2*CH_2pip_), 62.14 (CH_2_), 128.98 (2*CH_arom_), 129.16 (2*CH_arom_), 133.79 (C_arom_), 137.27 (C_arom_); FTIR (cm^−1^): 2962, 2917 (ν, CH), 2809, 2788, 2766, 2702, 2605, 2469 (ν, NH^+^), 1580 (ν, C=C_ar_); LC-MS (*m*/*z*): [C_12_H_19_N_2_]^+^ = 191.1543. 

*(5) 1-(2-Fluorobenzyl)piperazine hydrochloride*: white crystalline solid, m.p. = 165–166 °C; ^1^H NMR (ppm, CDCl_3_): 2.82 (4H, t, 2*CH_2pip_), 3.25 (4H, t, 2*CH_2pip_), 3.65 (2H, s, CH_2_), 7.01–7.14 (2H, m, 2*CH_arom_), 7.23–7.35 (2H, m, 2*CH_arom_), 9.58 (2H, bs, NH_2_^+^); ^13^C NMR (ppm, CDCl_3_): 43.49 (2*CH_2pip_), 49.27 (2*CH_2pip_), 54.93 (CH_2_), 115.37 and 115.66 (CH_arom_, C-F splitting), 123.31 and 123.49 (C_arom_, C-F splitting), 124.08 and 124.13 (CH_arom_, C-F splitting), 129.41 and 129.52 (CH_arom_, C-F splitting), 131.33 and 131.39 (CH_arom_, C-F splitting), 159.75 and 163.03 (C_arom_-F, C-F splitting); FTIR (cm^−1^): 2971, 2915 (ν, CH), 2821, 2791, 2705, 2677, 2654, 2627, 2502, 2492, 2474 (ν, NH^+^), 1587 (C=C_ar_); LC-MS (*m*/*z*): [C_11_H_16_FN_2_]^+^ = 195.1292.

*(6) 1-(4-Fluorobenzyl)piperazine hydrochloride*: white crystalline solid, m.p. = 170–171 °C; ^1^H NMR (ppm, CDCl_3_): 2.73 (4H, m, 2*CH_2pip_), 3.21 (4H, m, 2*CH_2pip_), 3.51 (2H, s, CH_2_), 6.95–7.01 (2H, m, 2*CH_arom_), 7.23 (2H, m, 2*CH_arom_), 9.40 (2H, bs, NH_2_^+^); ^13^C NMR (ppm, CDCl_3_): 43.55 (2*CH_2pip_), 49.28 (2*CH_2pip_), 61.56 (CH_2_), 115.17 (CH_arom_), 115.46 (CH_arom_), 130.40 (CH_arom_), 130.50 (CH_arom_), 132.63 (C_arom_), 160.59 and 163.84 (C_arom_-F, C-F splitting); FTIR (cm^−1^): 2916 (ν, CH), 2811, 2790, 2768, 2699, 2622, 2607, 2473 (ν, NH^+^), 1587 (C=C_ar_); LC-MS (*m*/*z*): [C_11_H_16_FN_2_]^+^ = 195.1292.

(products **IV**-**VI**). The reaction mixture was cooled down and the catalyst was filtered out (together with alternatively precipitated piperazine dihydrochloride). The filtrate was then placed into refrigerator overnight and precipitated piperazine dihydrochloride was filtered out. The solvent was then evaporated to dryness and the residue was recrystallized from isopropyl alcohol with addition of a charcoal to yield the pure product.

FT-IR spectra were measured on Bruker ATR Diamond using ATR measurement technique. NMR spectra were obtained on a Bruker Avance NMR III™ 300/75 MHz for the hydrogen/carbon spectrum using wideband probe BBFO. LC-MS analyses were performed on Agilent 1200 HPLC System and Agilent 6224 Accurate-Mass TOF mass spectrometer. Melting points were measured on a Boetius apparatus PHMK 05 (VEB Kombinat Nagema) with temperature gradient of 4 °C/min and their values are uncorrected. The reactions were monitored by TLC, which was conducted on aluminum plates TLC Silica gel 60 F_254_ by Merck. The detection methods and the eluents used for compounds are given in the procedure reports on syntheses of corresponding compounds [46,47,48,49,50]. Generally, the presence of substances was detected either by using UV lamp CAMAG (wavelength 254 nm or 366 nm) for compounds absorbing at the wavelengths, iodine vapors, or solution of ninhydrin (solution containing 2 g of ninhydrin, 100 mL of *n*-butanol and 20 mL of acetic acid) when NH_2_ or NH groups were present in the detected molecule.

## 4. Conclusions

In the article, we summarize recently obtained results and extend our research of monosubstituted piperazine derivatives with new findings concerning the microwave assisted acceleration of described synthetic reactions. A long-term study started with the invention of a new simple one-pot-one-step synthetic procedure leading to monosubstituted piperazines, and we then proceeded to draft a flow microwave reactor prototype which enables the more effective manufacturing of piperazine derivatives on a larger scale with prospects towards their potential commercial production.

Based on numerous experiments, we demonstrated that the described synthetic method based on a reaction of protonated piperazine can be further accelerated and can be thus made more efficient when microwave irradiation is involved. It was shown that the desired monosubstituted piperazine derivatives can be obtained in comparable yields and purity, but in significantly shorter times when microwave assisted synthetic techniques are employed. Although even the simple batch microwave process brought promising results, one should realize that a batch set-up can be regarded as a low cost and simple process only when a small volume of the reaction mixture is processed. When large-scale processing is considered, then investment into bigger and thus more expensive microwave devices is expected. To solve the problem, we adapted on an idea of a flow reactor consisting of a circulating system with only a small active part utilizing a small commercially available microwave unit. A consequent assembly of a functional prototype then proved its basic functionality. The proposed design of a flow reactor brings a lot of benefits, such as variability, speed, and efficiency, all for a low price, as it can be constructed using cheap and easily available parts and materials. This also comes before the consideration that a flow microwave reactor equipped with a catalytic bed can be employed in the synthesis/manufacturing of a variety of different products, as it can be operated in three working modes depending on a type of reaction. Thus, our research is interesting for a wider sphere of chemists and manufacturers. 

## 5. Patents

Procedures for a direct *N*-monosubstitution of piperazine are subject of a patent protection under the numbers CZ 305854 (2016), CZ 305317 (2015), CZ 304520 (2014). The description of supported metal catalysts can be found under patent number CZ 305277 (2015). The technology of a batch microwave process and of a flow microwave process including a flow reactor is protected as a classified know-how of Masaryk University. The flow reactor with microwave unit and/or catalytic bed is protected as a utility model under number UV 32201 (2018). The flow ultrasonic reactor is protected as a utility model under number UV 24590 (2012).

## Figures and Tables

**Table 1:** Results of syntheses of monosubstituted piperazine derivatives using different synthetic techniques.

Reactant (R)	Molar Ratio ^1^	Catal.	Proc. ^2^	Time (hr)	Product	Aver. Yield ^3^ (%)
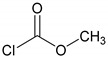	-: 1.3	Cu(I)	A	24	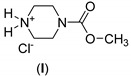	70
	-	-	B ^4^	-		-
	-: 1.1	Ce(III)	C ^4^	0.58		61
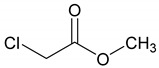	1: 1.1	-	A	19	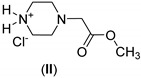	57
	1: 1.2	Ce(III)	A	8		61
	1: 1.2	Cu(II)	B	6		54
	1: 1.2	Cu(II)	C	2.17		64
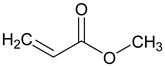	1: 2.2	-	A	16	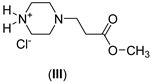	62
	1: 2.6	Ce(III)	A	7		60
	1: 2		B	4		50
	1.2: 2.7		C	0.17		61
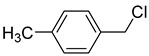	0.2: 1.2	Cu(II)	A	8	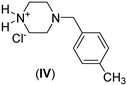	71
	0.2: 1.1		B	1.83		72
	-		C	-		-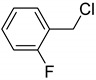	1: 1.1	Cu(II)	A	13	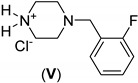	84
	0.5: 1.1		B	2.5		67
	-		C	-		-
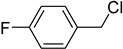	0.5: 1.1	Cu(II)	A	14	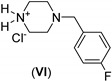	88
	0.5: 1.1		B	1		69
	-		C	-		-

^1^ Molar ratios are listed as piperazine. 2HCl: reactant and are always related to 1 mol of anhydrous piperazine. ^2^ Proc. **A** follows literature method [46,47,48,49,50], proc. **B** (batch) and **C** (flow) proceed under MW irradiation. ^3^ Average yield of a recrystallized product with respect to anhydrous piperazine excepting reactions of methyl acrylate which is related to piperazine monohydrochloride. ^4^ The reaction proceeds at room temperature and thus only a simple flow mode using catalyst was applied.

## Supplementary Materials

The following are available online. Spectra of above-mentioned products obtained by different analytical techniques.
