# Supplementary material for: Simplified Procedure for General Synthesis of Monosubstituted Piperazines—From a Batch Reaction Vessel to a Flow (Microwave) Reactor
Source: Molecules. 2020 May 6;25(9):2168. doi: 10.3390/molecules25092168 (PMC7249161; doi:10.3390/molecules25092168)
Supplement: Supplementary file 1 [file molecules-25-02168-s001.zip › LC-MS/Product III - LC-MS_watermark.pdf]

Sample name: MP0003

Summary formula: $C_8H_{17}ClN_2O_2$

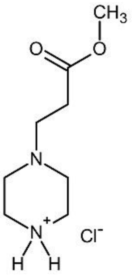

LC:

Agilent 1200 HPLC System

Chromatographic column: Phenomenex Luna Omega PS C18, 150 x 4.6 mm, 3µm

Flow rate: 1 mL/min

Injection volume: 2 µL of aqueous sample (c = 100 µg/mL)

Gradient elution:

Gradient elution:	0 – 13 min	100 – 85% 30 mM ammonium acetate (0 – 15% ACN)
	13 – 16 min	85 – 35% 30 mM ammonium acetate (15 – 65% ACN)
	16 – 17 min	35 – 0% 30 mM ammonium acetate (65 - 100% ACN)

MS:

Agilent 6224 Accurate-Mass TOF mass spectrometer

MS conditions: APCI positive, N_2 flow 7 L/min, gas temperature 325°C, nebulizer 45 psig, vaporizer 200°C, fragmentor 20 V

$[C_8H_{17}N_2O_2]^+$ = 173.1285

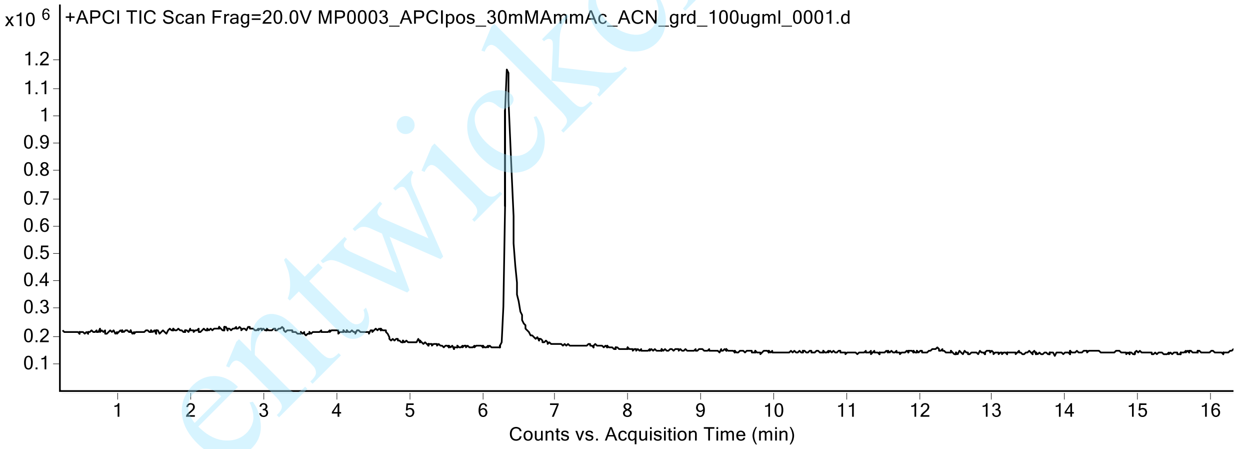

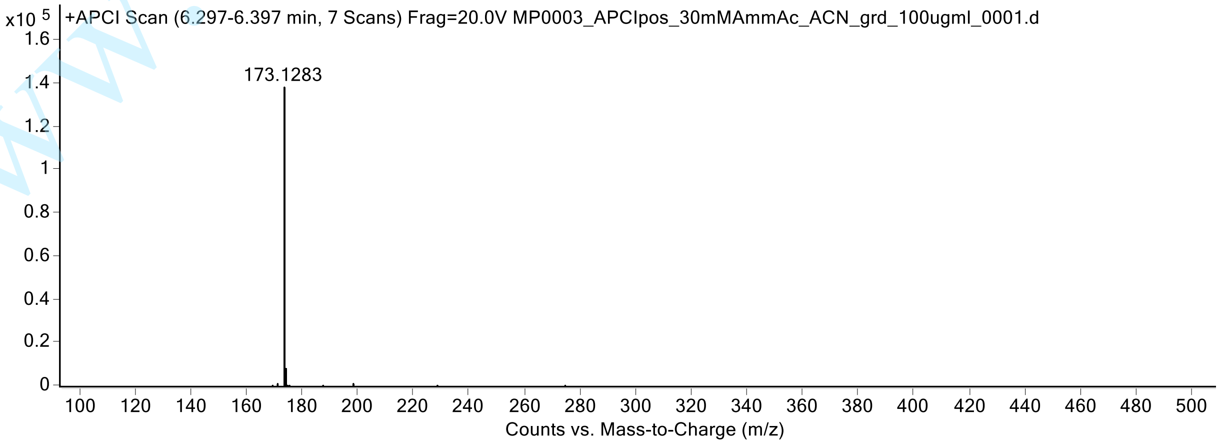

www.entwickchemicals.com
