# Supplementary figures and images for: Simplified Procedure for General Synthesis of Monosubstituted Piperazines—From a Batch Reaction Vessel to a Flow (Microwave) Reactor
Source: Molecules. 2020 May 6;25(9):2168. doi: 10.3390/molecules25092168 (PMC7249161; doi:10.3390/molecules25092168)

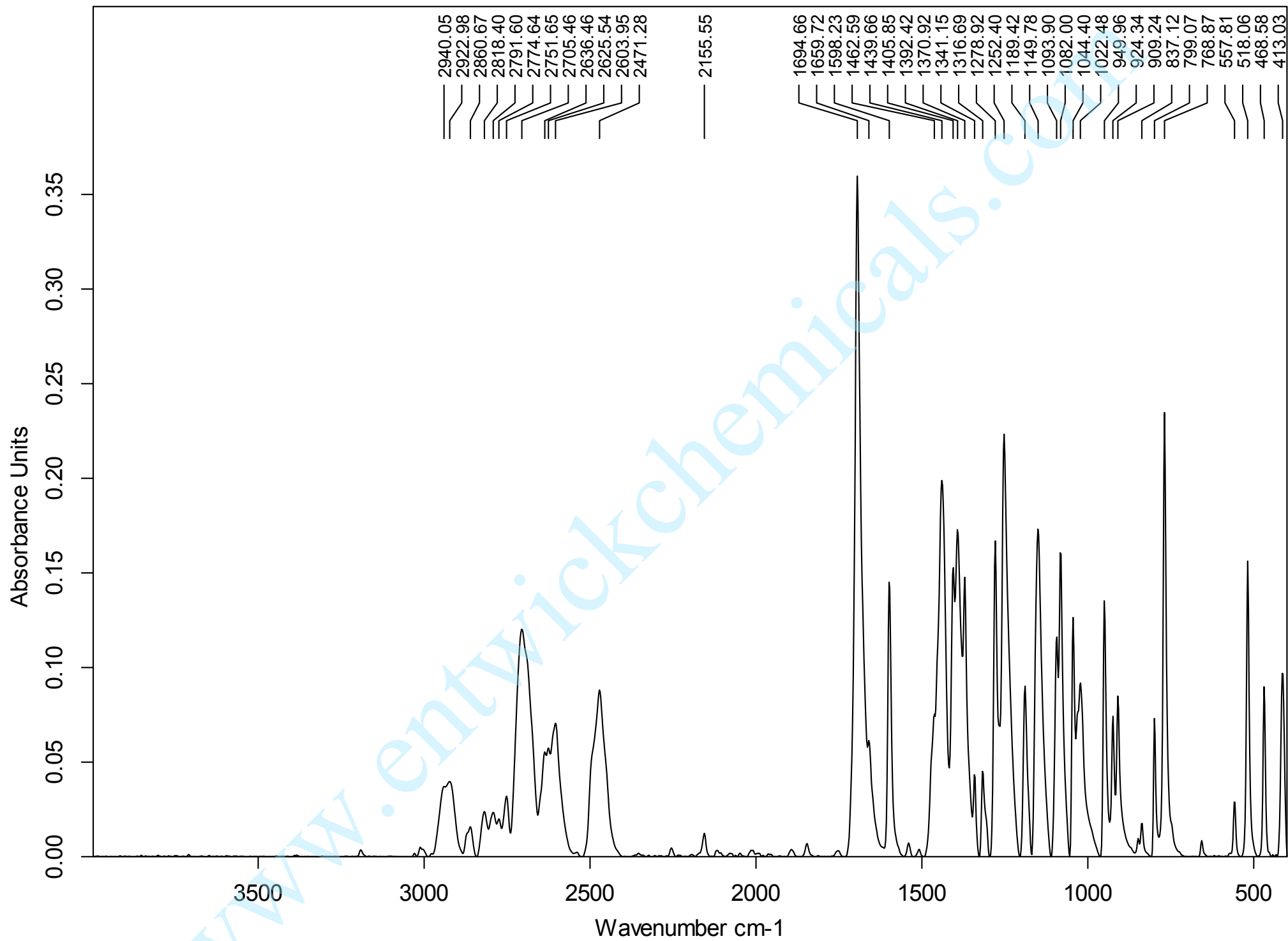

Supplement: Supplementary file 1 [file molecules-25-02168-s001.zip › FTIR/Product I - FTIR_watermark.pdf]

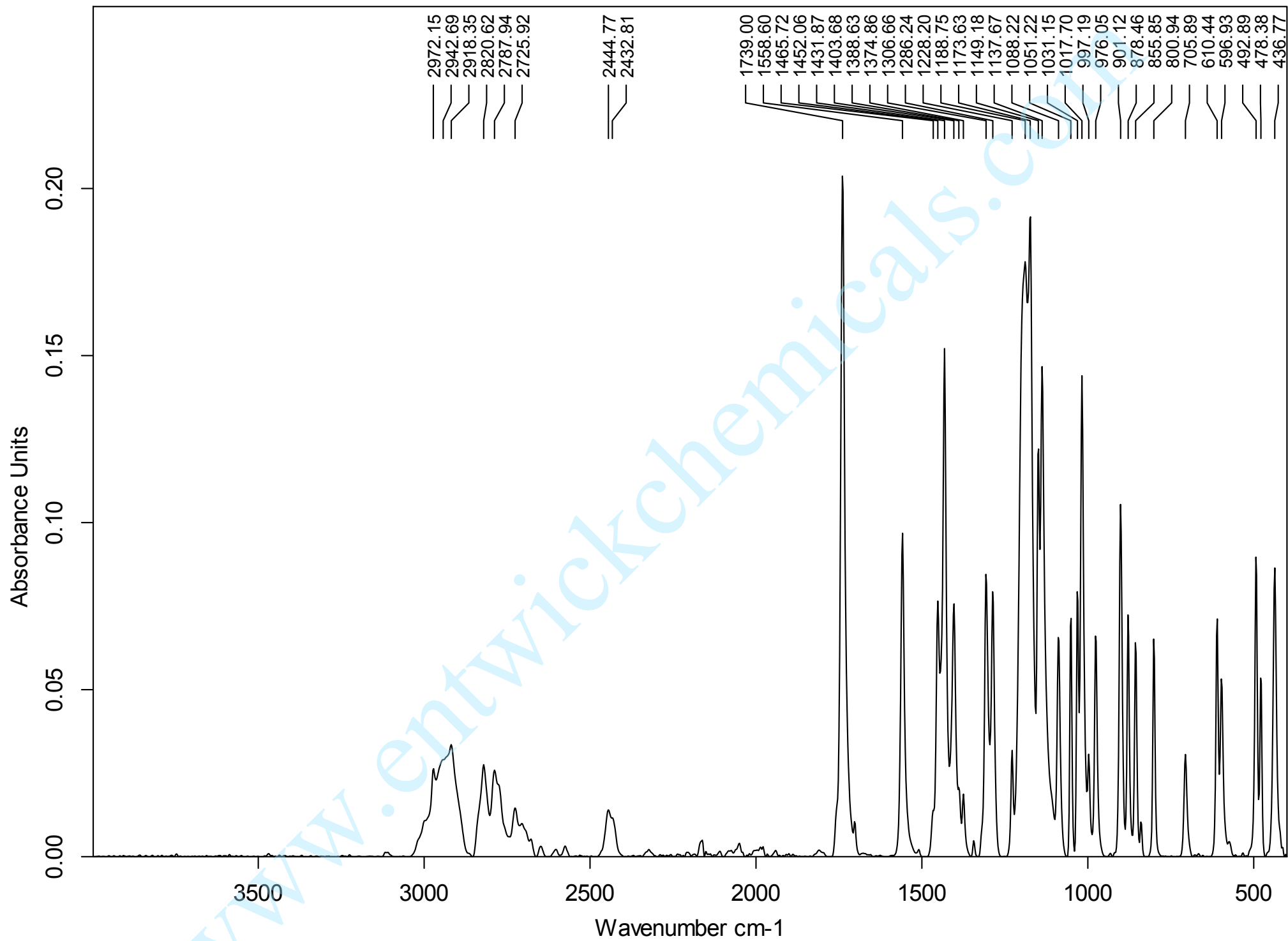

Supplement: Supplementary file 1 [file molecules-25-02168-s001.zip › FTIR/Product II - FTIR_watermark.pdf]

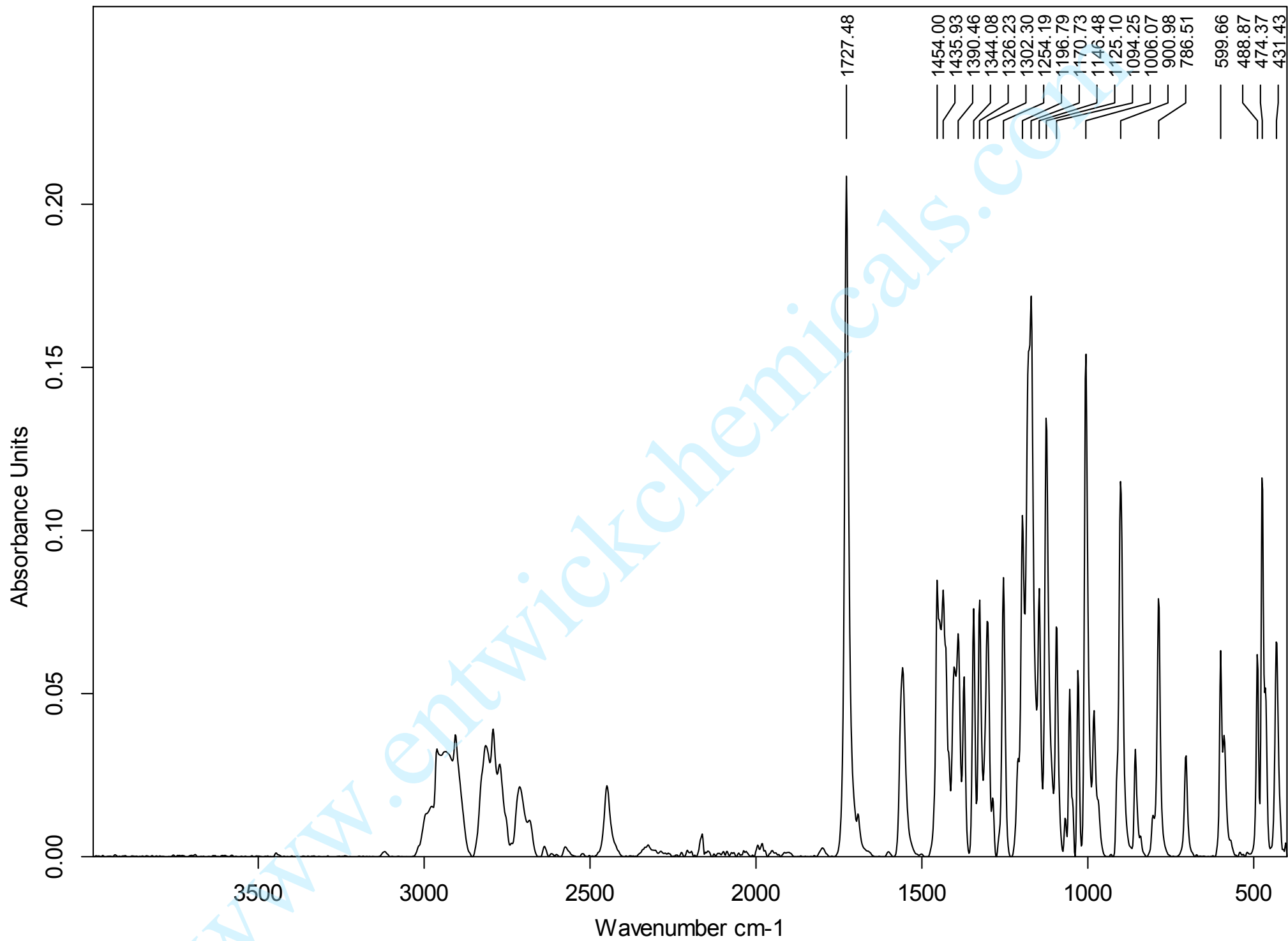

Supplement: Supplementary file 1 [file molecules-25-02168-s001.zip › FTIR/Product III - FTIR_watermark.pdf]

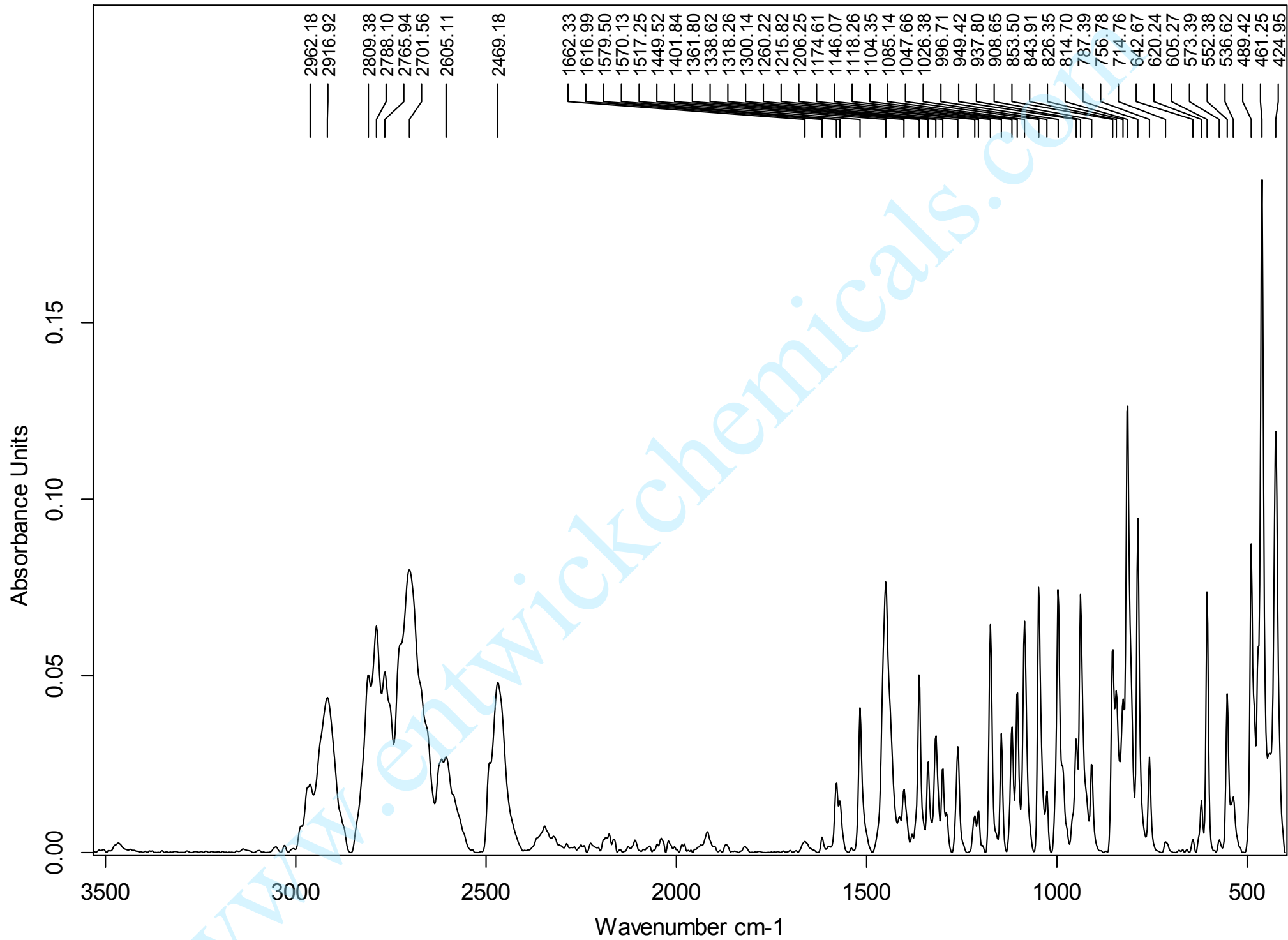

Supplement: Supplementary file 1 [file molecules-25-02168-s001.zip › FTIR/Product IV - FTIR_watermark.pdf]

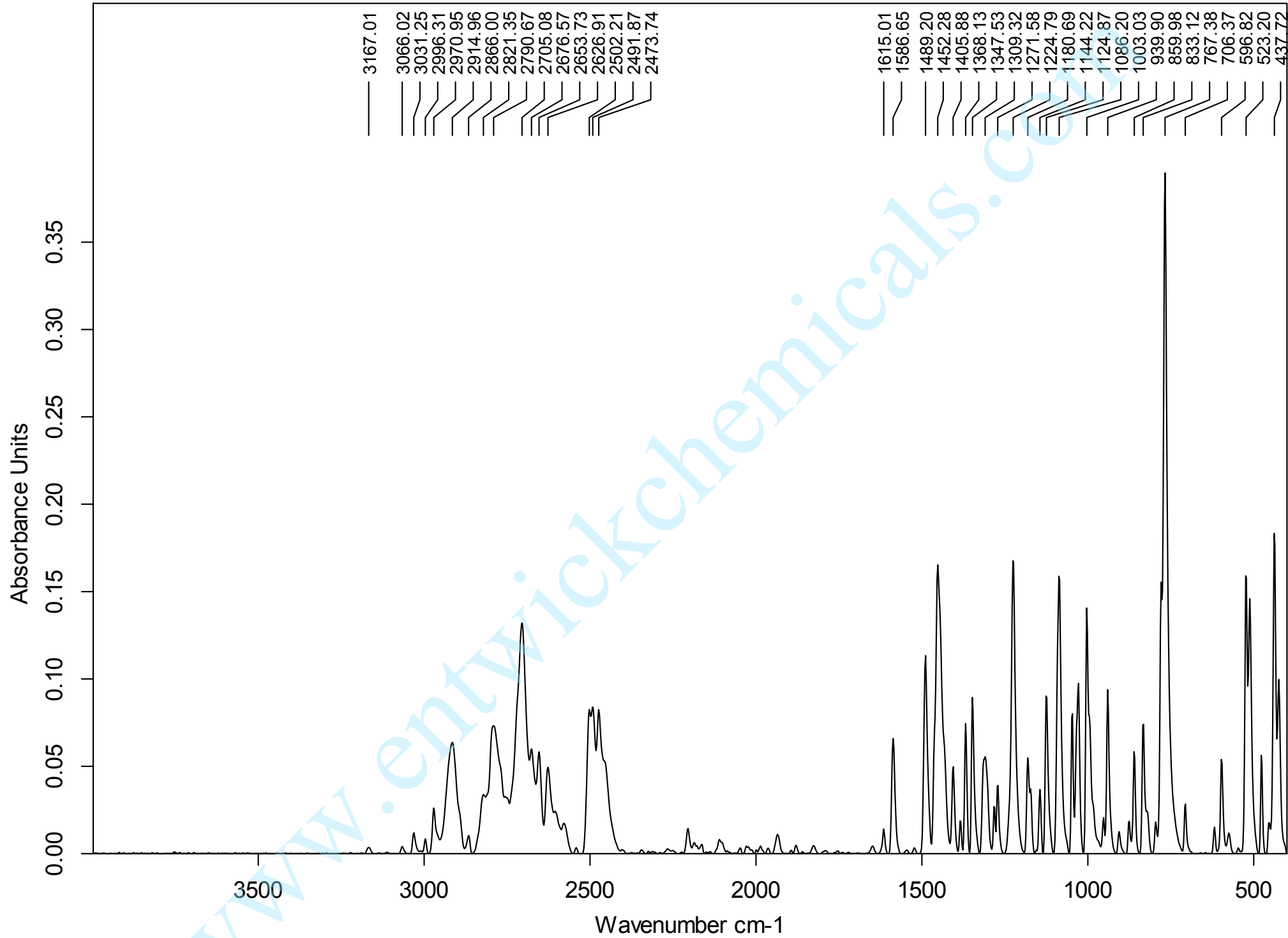

Supplement: Supplementary file 1 [file molecules-25-02168-s001.zip › FTIR/Product V - FTIR_watermark.pdf]

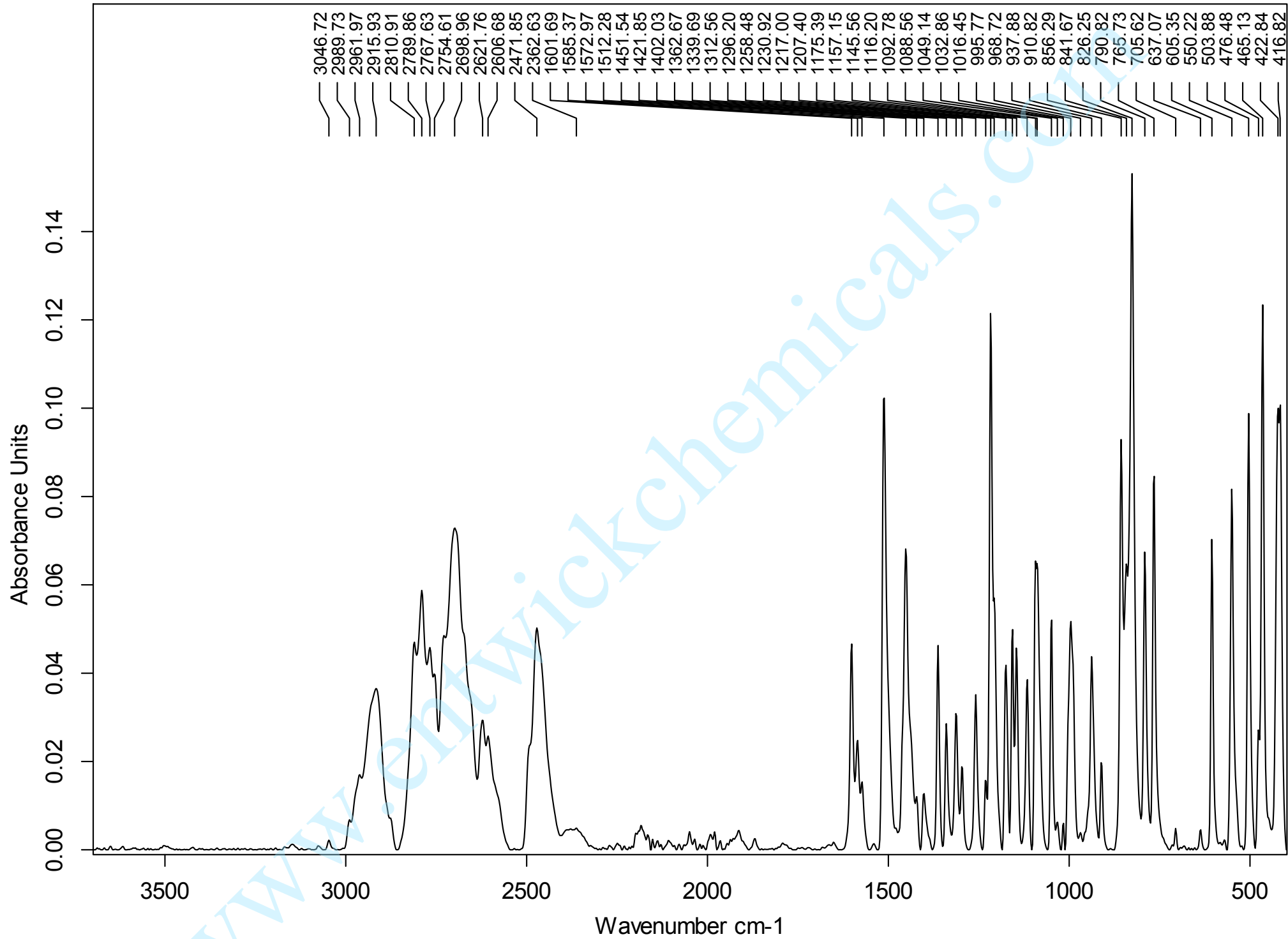

Supplement: Supplementary file 1 [file molecules-25-02168-s001.zip › FTIR/Product VI - FTIR_watermark.pdf]

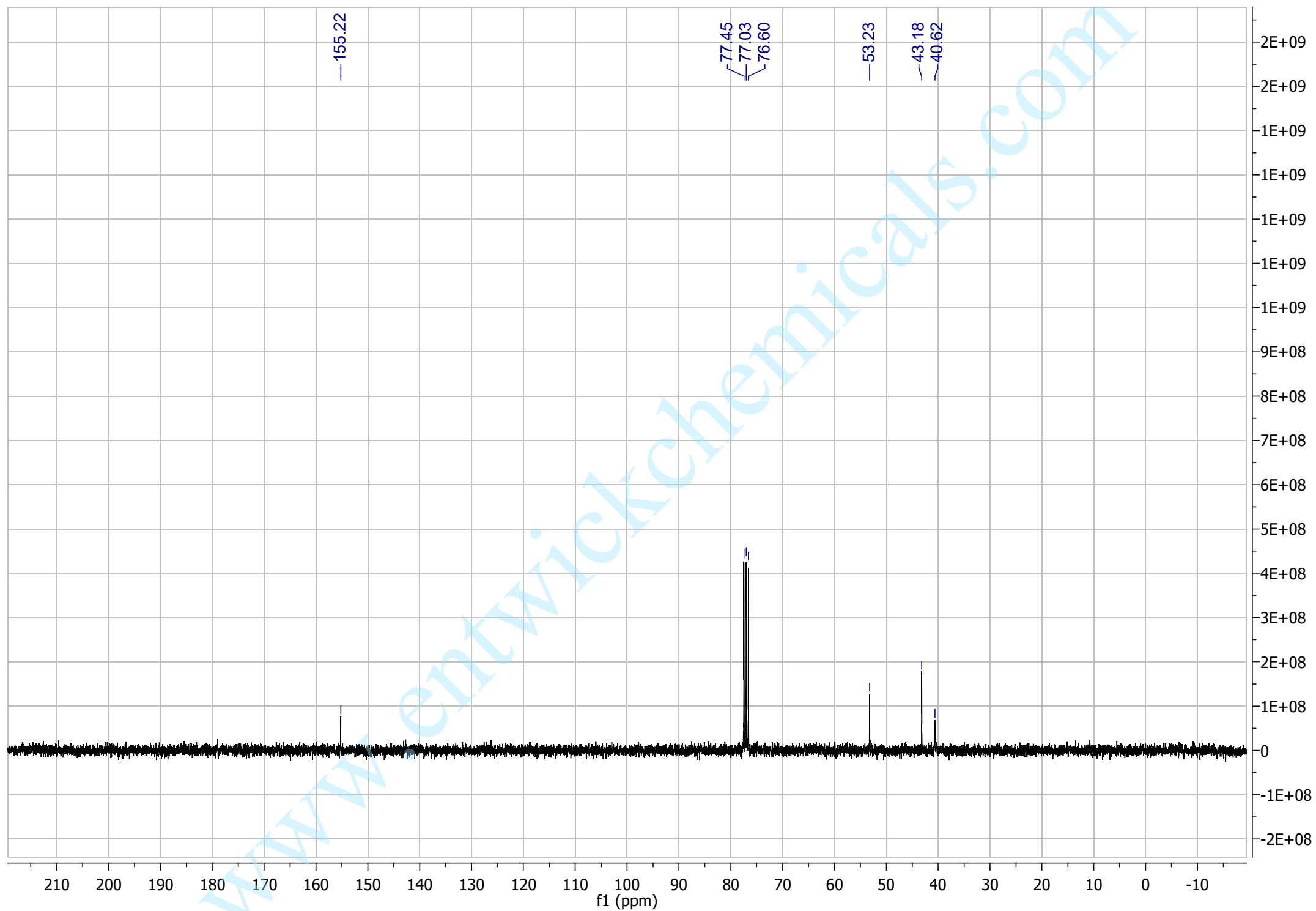

Supplement: Supplementary file 1 [file molecules-25-02168-s001.zip › NMR/Product I - 13C_NMR_CDCl3_watermark.pdf]

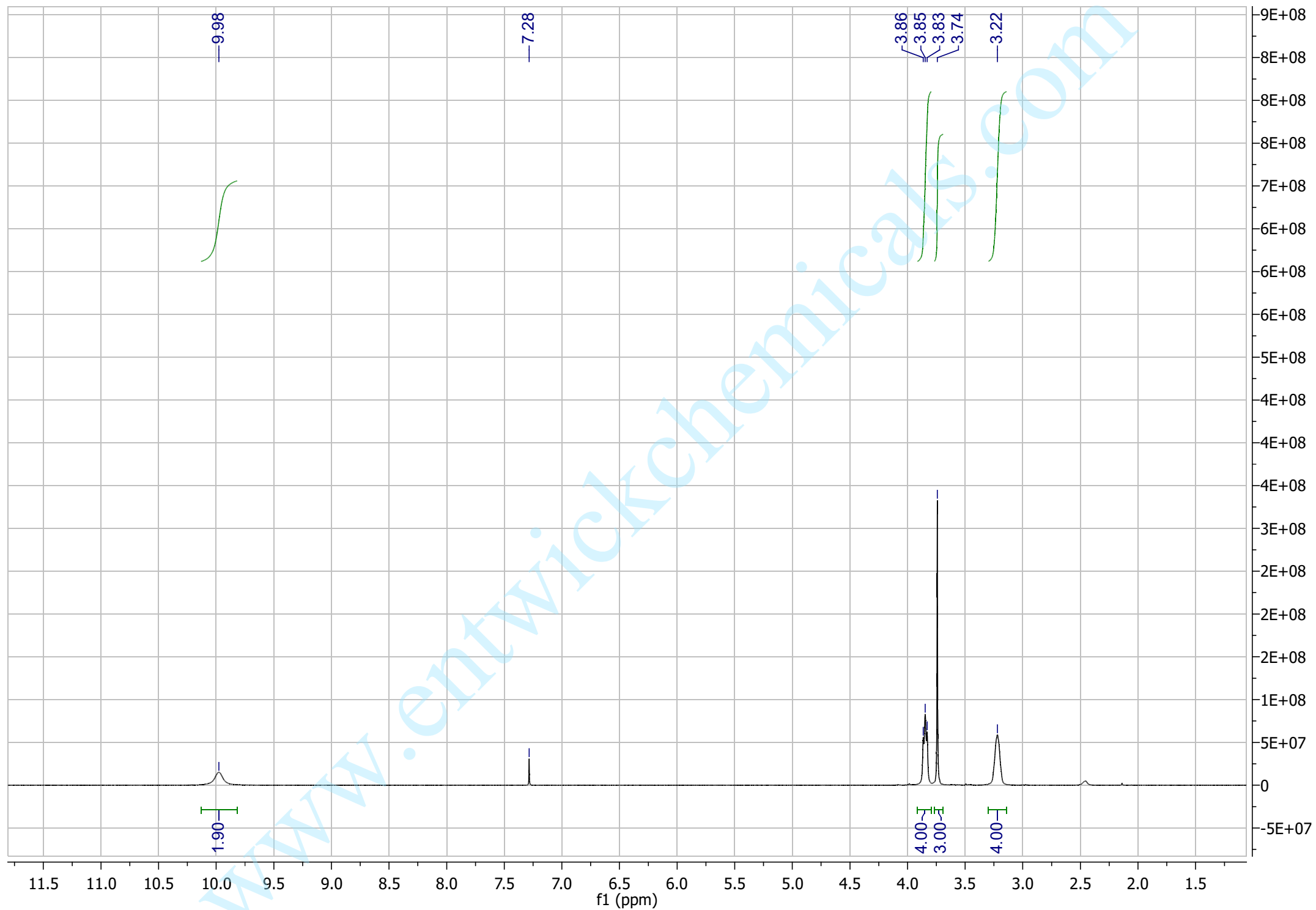

Supplement: Supplementary file 1 [file molecules-25-02168-s001.zip › NMR/Product I - 1H_NMR_CDCl3_watermark.pdf]

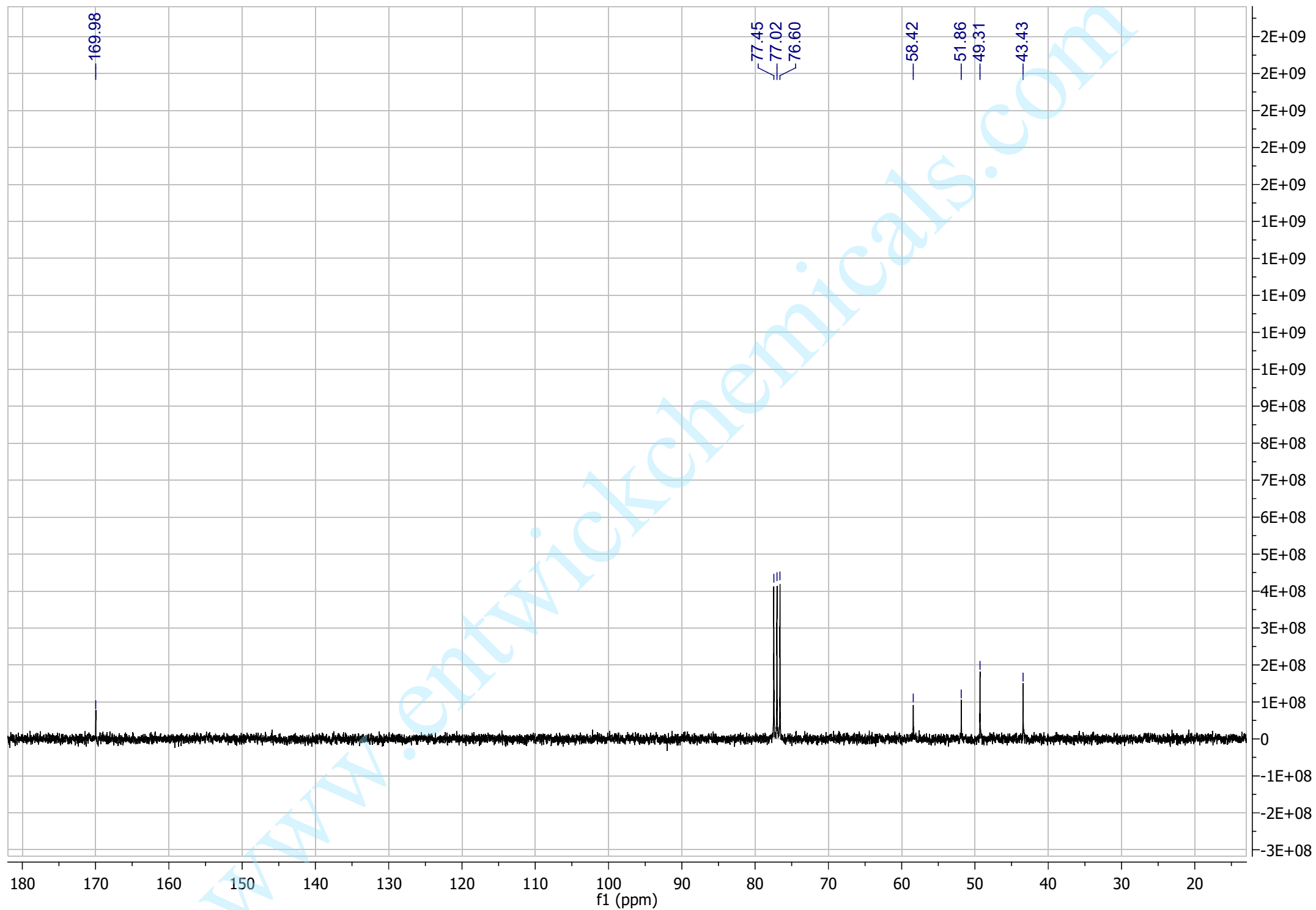

Supplement: Supplementary file 1 [file molecules-25-02168-s001.zip › NMR/Product II - 13C_NMR_CDCl3_watermark.pdf]

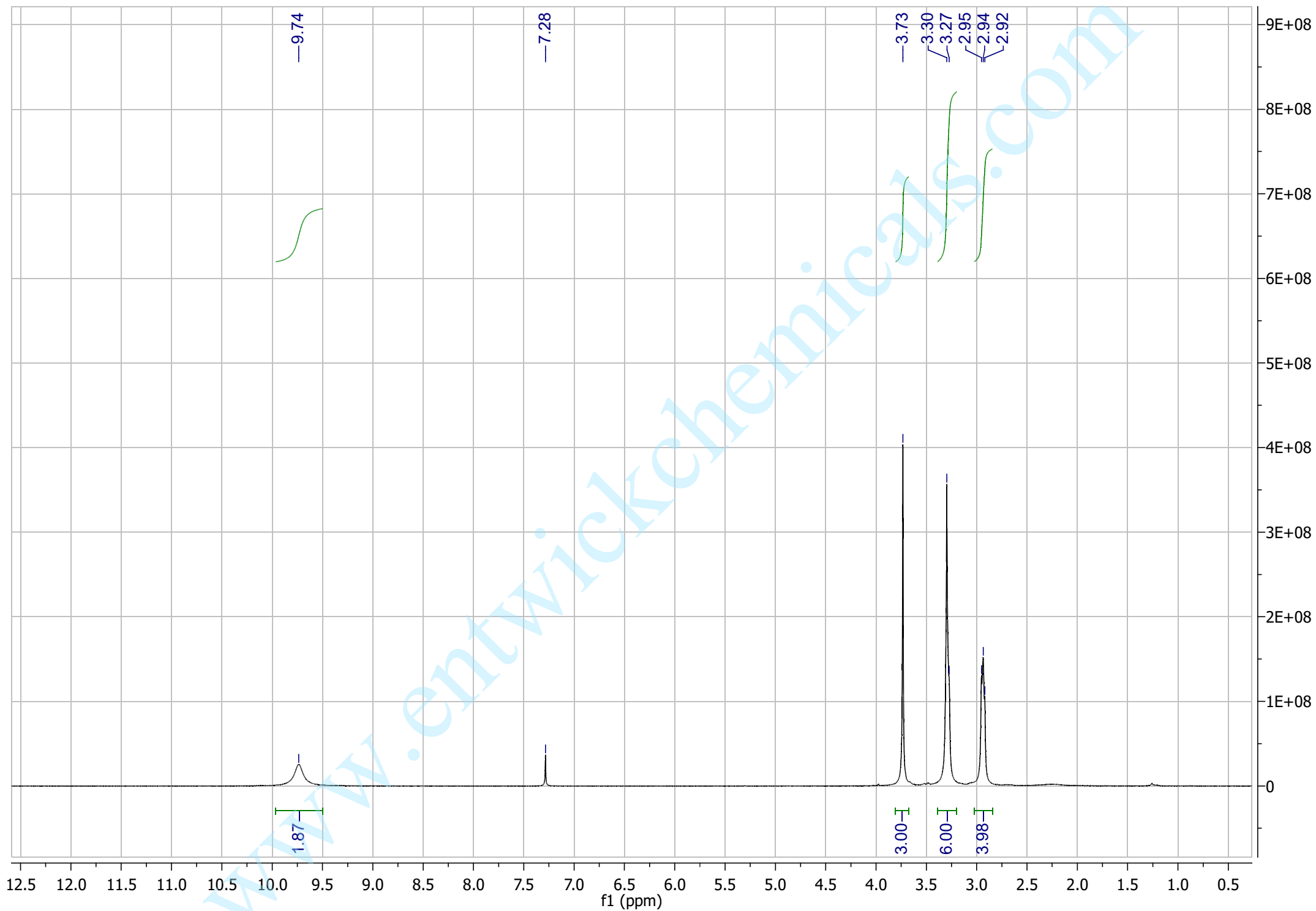

Supplement: Supplementary file 1 [file molecules-25-02168-s001.zip › NMR/Product II - 1H_NMR_CDCl3_watermark.pdf]

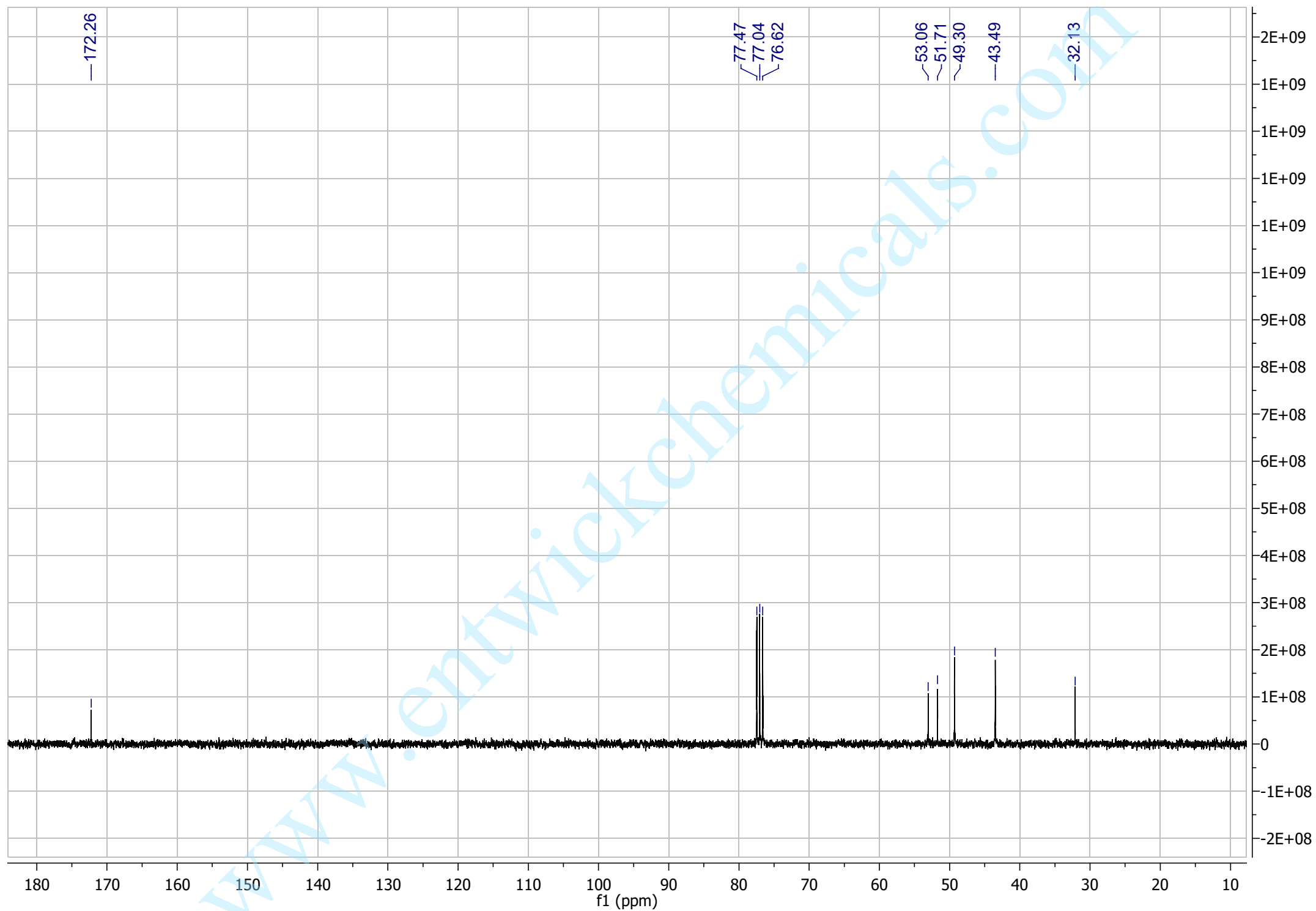

Supplement: Supplementary file 1 [file molecules-25-02168-s001.zip › NMR/Product III - 13C_NMR_CDCl3_watermark.pdf]

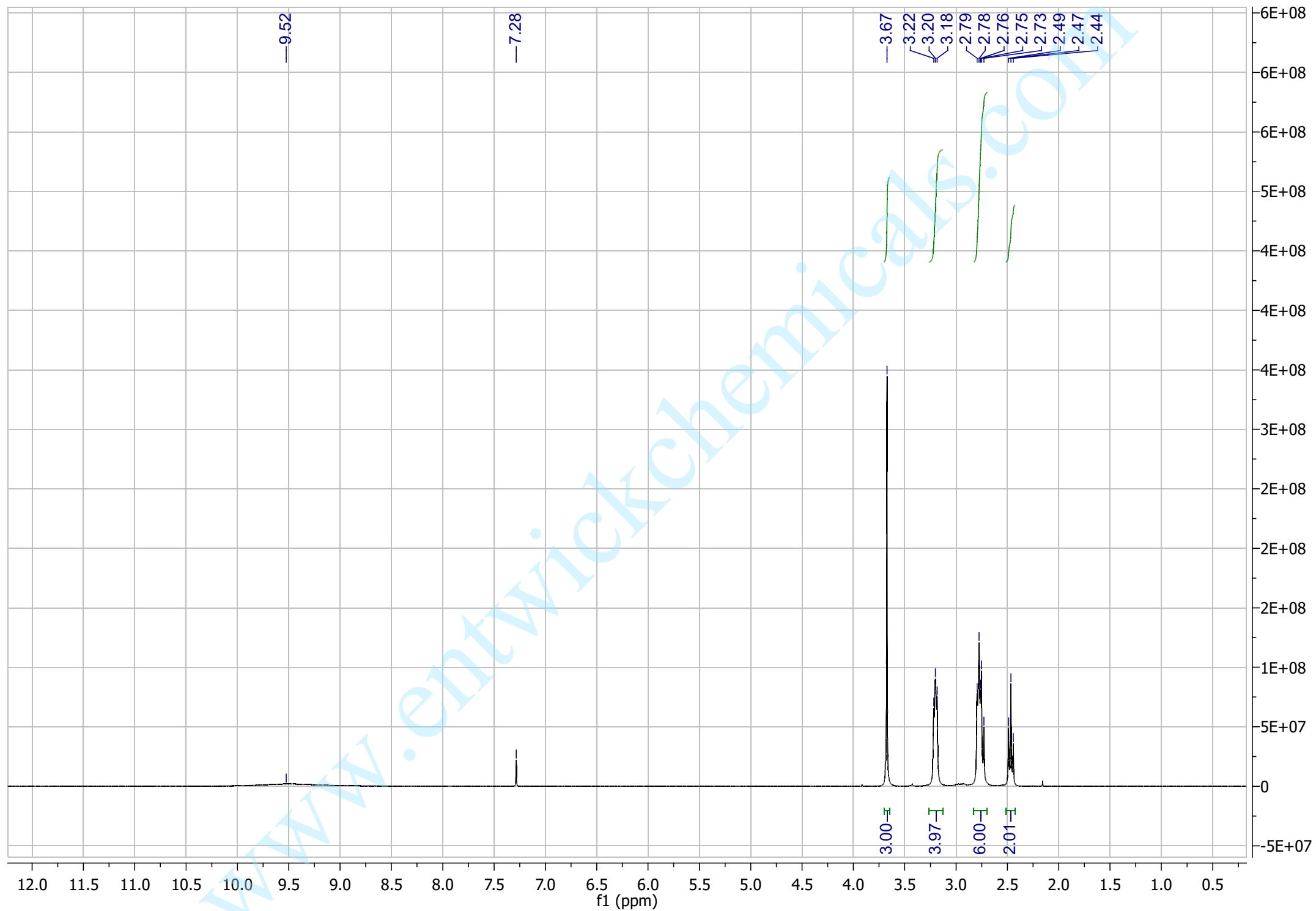

Supplement: Supplementary file 1 [file molecules-25-02168-s001.zip › NMR/Product III - 1H_NMR_CDCl3_watermark.pdf]

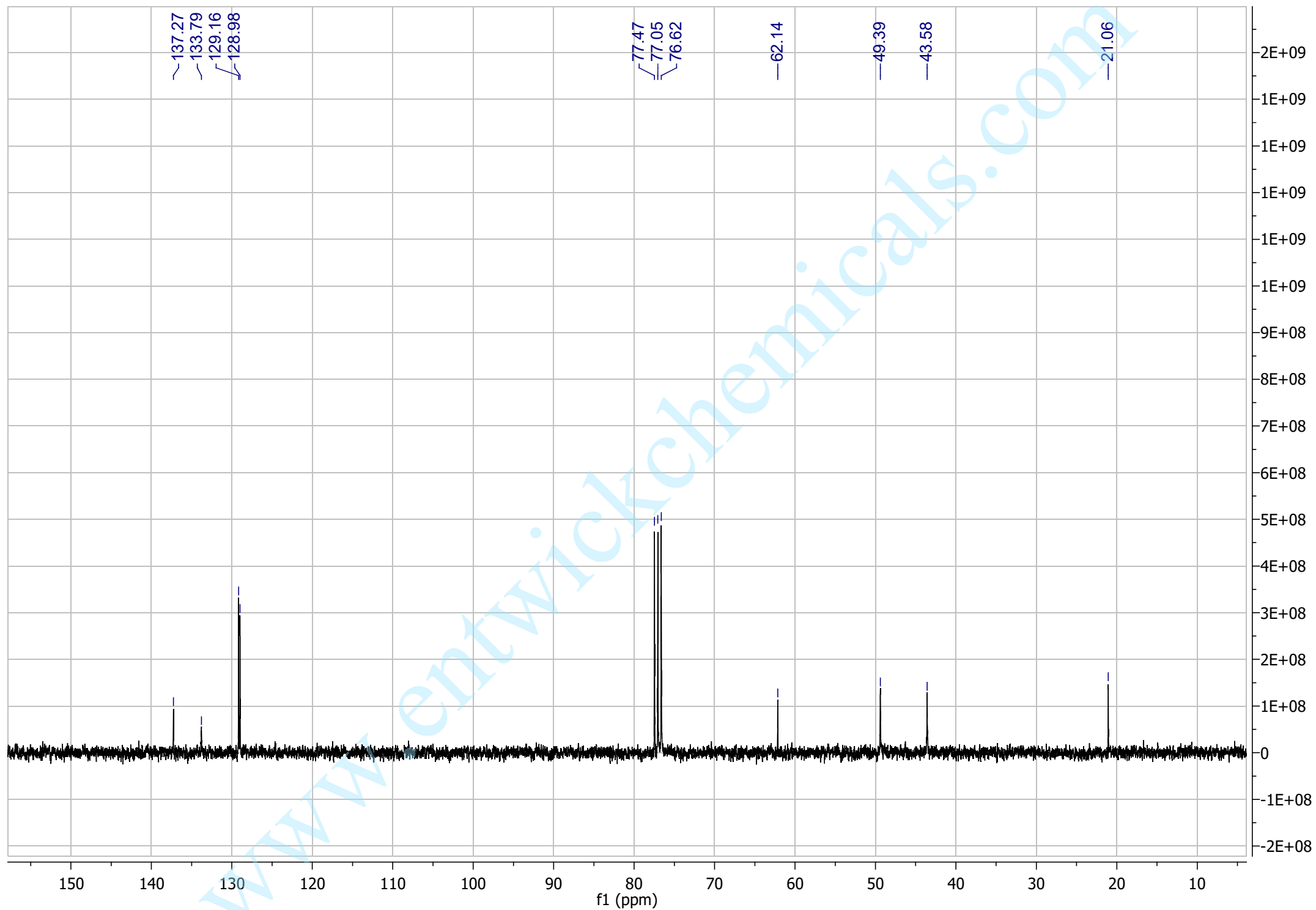

Supplement: Supplementary file 1 [file molecules-25-02168-s001.zip › NMR/Product IV - 13C_NMR_CDCl3_watermark.pdf]

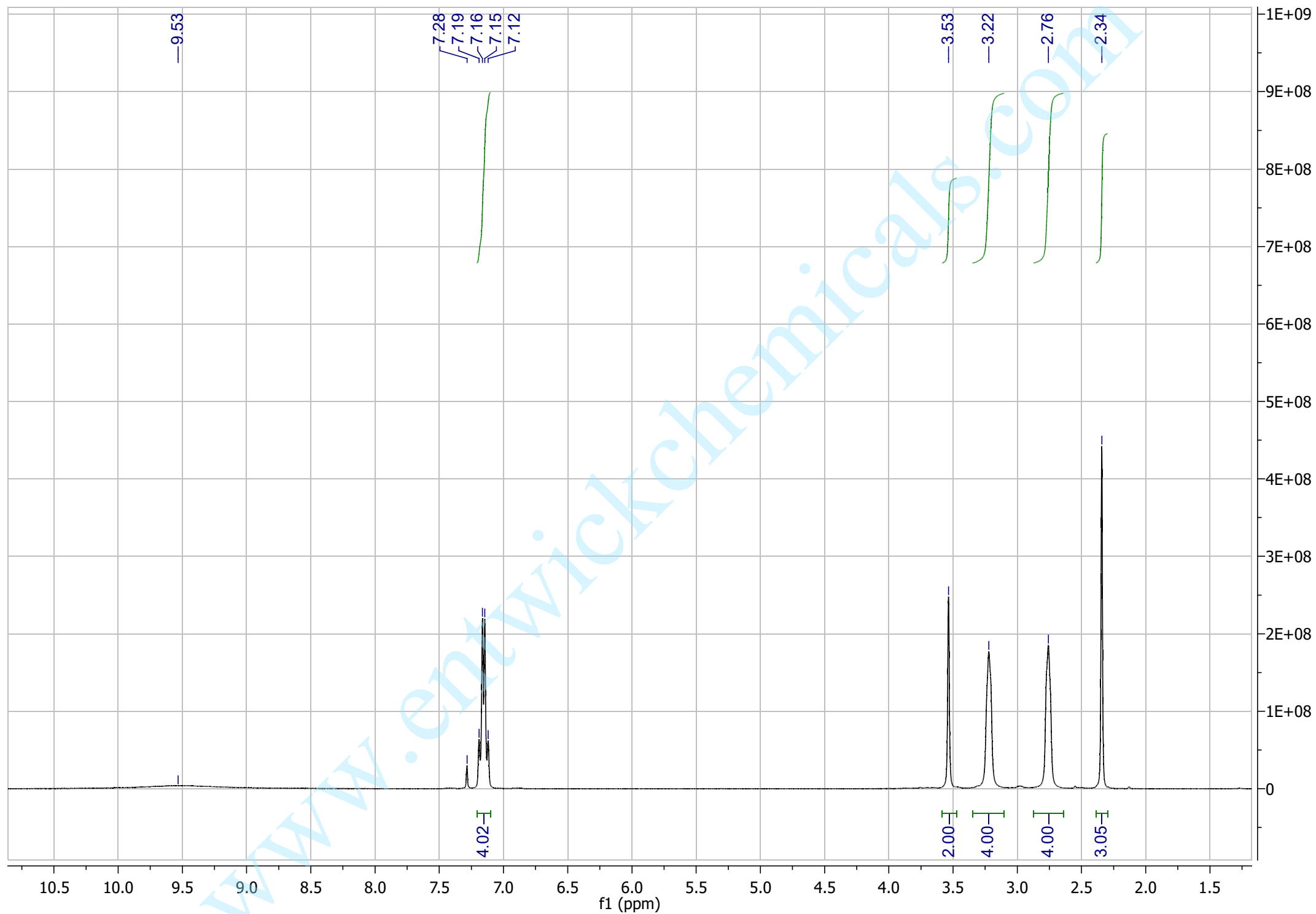

Supplement: Supplementary file 1 [file molecules-25-02168-s001.zip › NMR/Product IV - 1H_NMR_CDCl3_watermark.pdf]

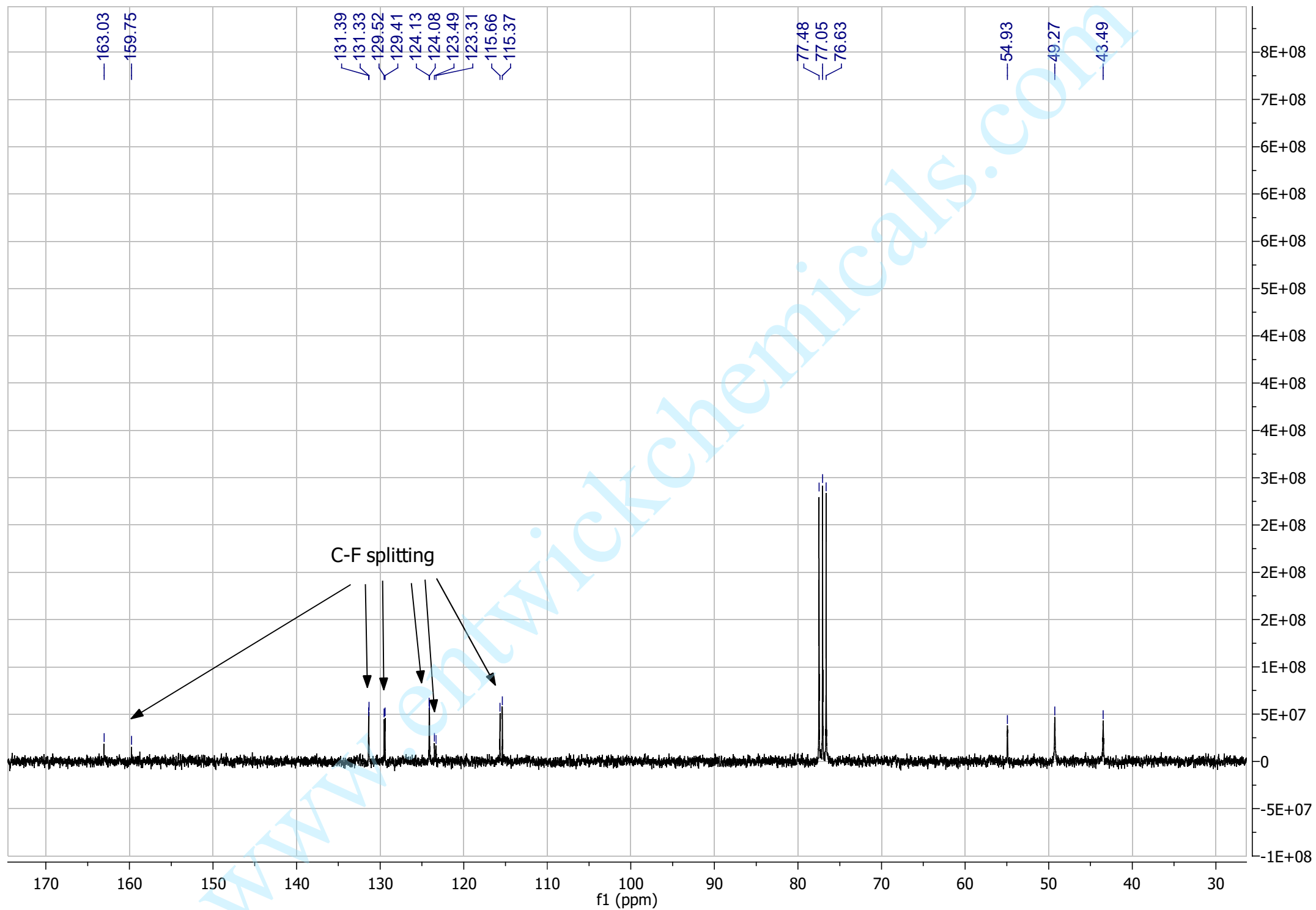

Supplement: Supplementary file 1 [file molecules-25-02168-s001.zip › NMR/Product V - 13C_NMR_CDCl3_watermark.pdf]

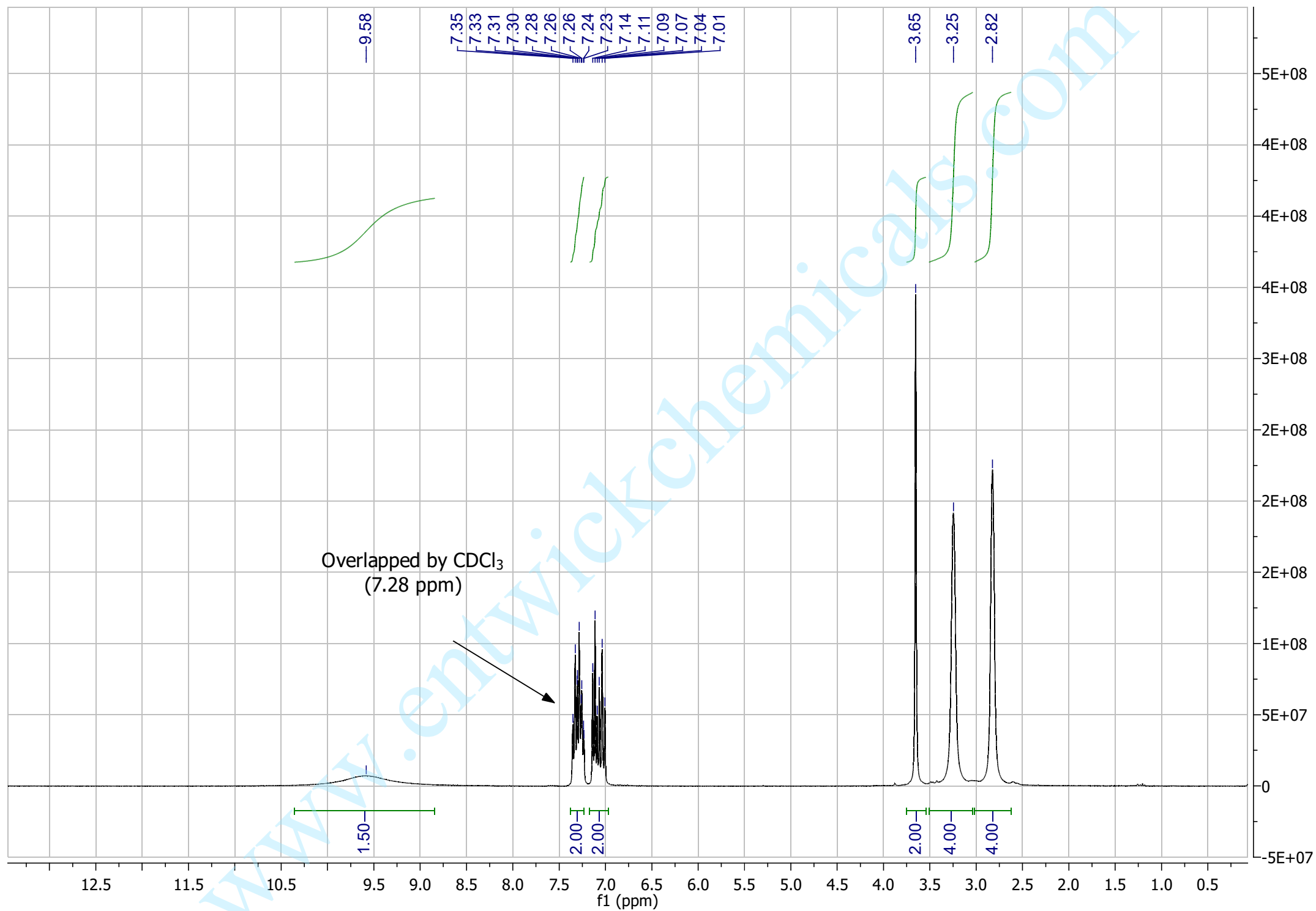

Supplement: Supplementary file 1 [file molecules-25-02168-s001.zip › NMR/Product V - 1H_NMR_CDCl3_watermark.pdf]

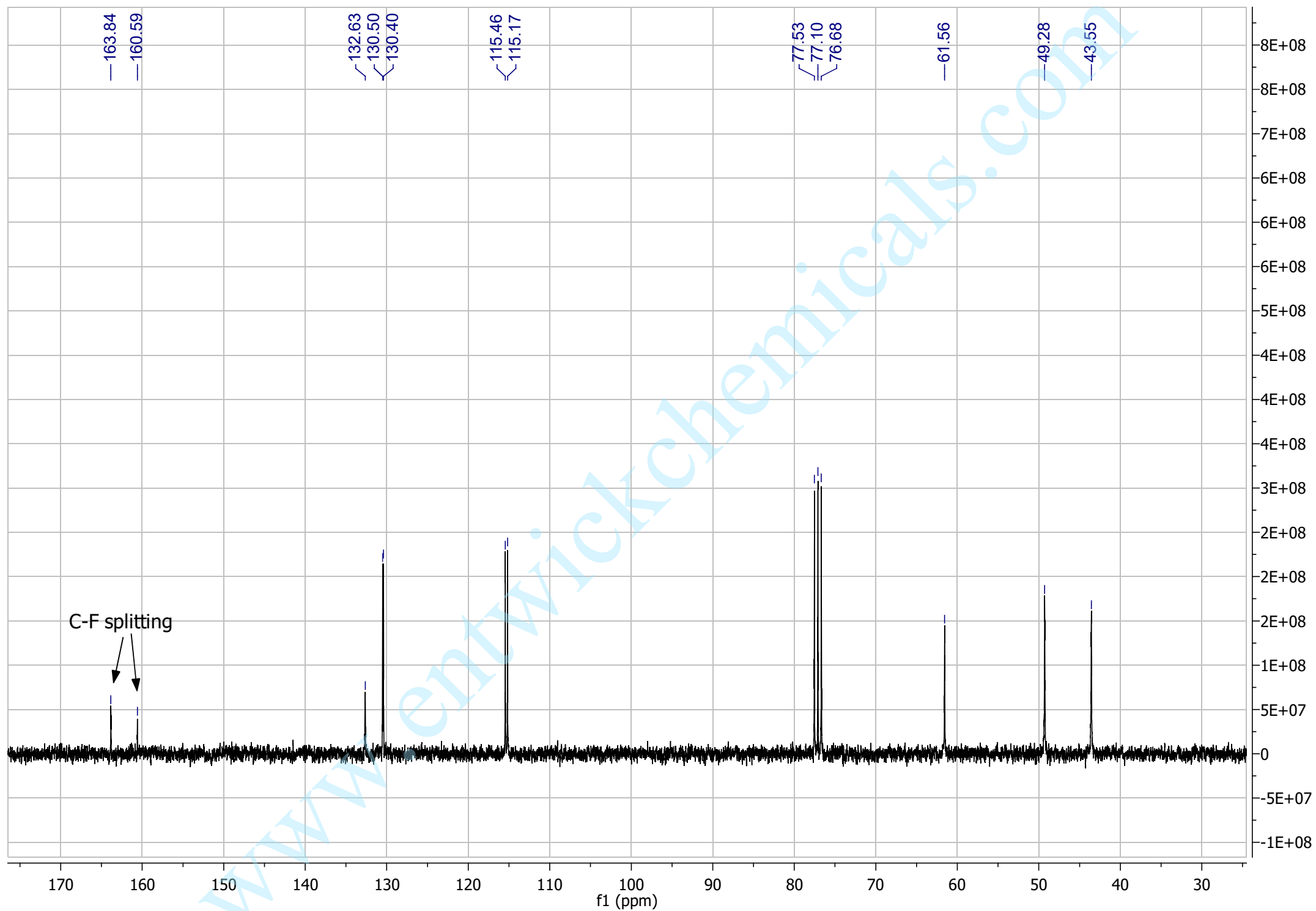

Supplement: Supplementary file 1 [file molecules-25-02168-s001.zip › NMR/Product VI - 13C_NMR_CDCl3_watermark.pdf]

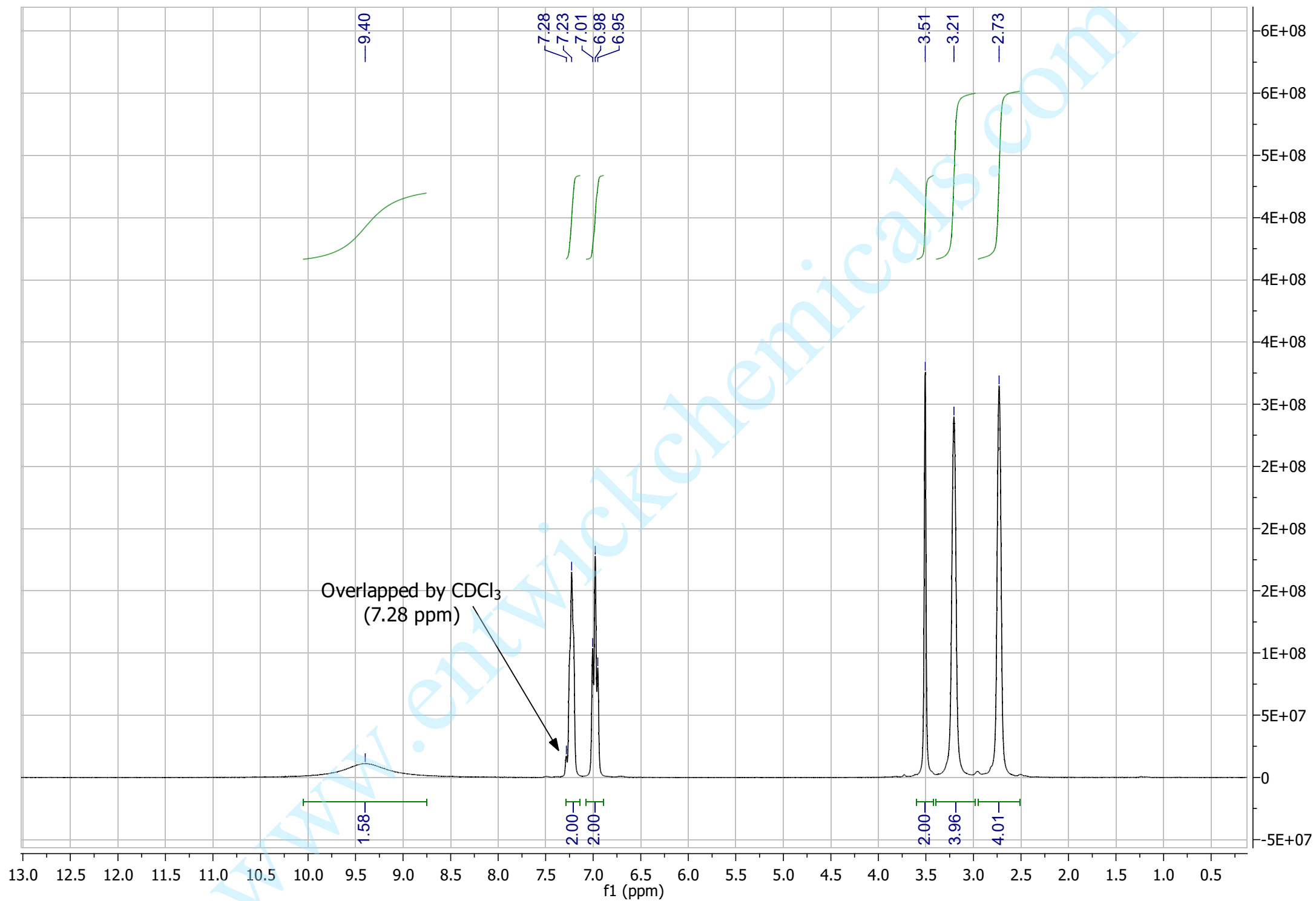

Supplement: Supplementary file 1 [file molecules-25-02168-s001.zip › NMR/Product VI - 1H_NMR_CDCl3_watermark.pdf]
